# Engineering Design Strategies for Boosting Photocatalytic Activity: Theory-to-Data-Driven Perspective

**DOI:** 10.3390/ma19071472

**Published:** 2026-04-07

**Authors:** Wilian Jesús Pech-Rodríguez, Nihat Ege Şahin, Gladis Guadalupe Suarez-Velázquez

**Affiliations:** 1 Department of Master’s Program, Engineering Science, Polytechnic University of Victoria, Ciudad Victoria 87138, Tamaulipas, Mexico; 2 Department of Energy Conversion and Storage, Technical University of Denmark, 2800 Kongens Lyngby, Denmark; 3 Department of Energy Engineering, Polytechnic University of Altamira, Nuevo Libramiento Altamira Km. 3, Santa Amalia, Altamira 89602, Tamaulipas, Mexico

**Keywords:** photocatalysts, materials engineering, dye degradation, machine learning-assisted design, theoretical modeling

## Abstract

Photocatalysts have emerged as a promising approach for the treatment of contaminated water, particularly for the removal of dyes and pharmaceutical residues that pose risks to human health. In addition, they can be employed for the generation of chemical fuels such as H_2_ and oxidizers such as H_2_O_2_, which have been proposed as sustainable energy carriers to reduce reliance on fossil fuels. The first part of this brief review provides a detailed overview of the fundamental concepts of photocatalysis, including reaction pathways and reported mechanisms. The second part explores the main design strategies for enhancing photocatalytic performance, including morphology control and structural modification. Then, the third section highlights the benefits of theoretical modeling, including first-principles calculations and molecular simulations. The document culminates with a section on challenges and future perspectives, highlighting major issues in photocatalyst development such as large-scale synthesis, material stability, and reusability. This brief review is intended to provide young researchers with a concise understanding of the most effective strategies for enhancing photocatalytic performance, as well as the mechanisms influencing morphology and structural parameters. This work presents an integrated framework linking synthesis strategies, particle growth mechanisms, multidimensional nanostructures, in situ and operando characterization, and computational modeling to guide the rational design of next-generation photocatalysts.

## 1. Introduction

Photocatalytic materials are central to the development of low-carbon technologies that address global energy and environmental challenges [1,2,3]. By harnessing light energy to drive chemical transformations, these materials offer a route for sustainable fuel production and environmental remediation [4,5]. Among the most widely studied photocatalysts are zinc oxide (ZnO) and titanium dioxide (TiO_2_), due to their chemical stability, low toxicity, abundance, and relatively low cost [6]. These materials are characterized by their wide band gap, ~3.2–3.3 eV for ZnO and ~3.0–3.2 eV for TiO_2_ [7,8]. Both semiconductors have been widely applied in environmental remediation, achieving >80–95% degradation of model pollutants within 30–120 min of irradiation [9], and also in hydrogen production (100–1000 μmol h^−1^ g^−1^) and CO_2_ reduction [10]. Nevertheless, their overall efficiency remains limited by rapid electron-hole recombination. One of the main obstacles for photocatalytic materials is their low stability and declining performance in light harvesting under operational conditions, caused by multiple competing pathways or the presence of intermediates [11,12]. Consequently, several strategies have been proposed to maintain performance, including the synthesis of multi-element materials, doping, polymer incorporation, and the use of other advanced compounds [13,14,15]. Among these strategies, the construction of heterojunctions has emerged as one of the most effective approaches to enhance photocatalytic activity [16]. Heterojunctions formed between two or more semiconductors, such as type-II, Z-scheme, or S-scheme architectures, promote spatial separation of photogenerated electrons and holes by creating internal electric fields at the interface. In many cases, heterojunction photocatalysts exhibit 2–10 times higher degradation rates of organic pollutants compared with single-component materials. For example, Liu et al. reported that an Ag-modified TiO_2_/ZnO heterojunction showed an 8-fold increase in photocurrent [17]. Furthermore, heterostructures can improve photostability and catalytic durability, maintaining over 80–90% of their initial activity after 5–10 catalytic cycles [18]. Engineering design represents a feasible approach to enhance the photoactivity of developed materials, as it allows for the consideration from the outset of morphological, chemical, and surface effects on the photocatalytic process [19,20]. Furthermore, this approach can be supported by theoretical calculations, such as first-principles modeling, or by the application of advanced intelligent algorithms to elucidate effects at the atomistic scale [21,22].

Photocatalysts play a significant societal role, as they can be used to treat dyes, heavy metals, and pesticides present in industrial wastewater and agricultural effluents, which pose serious environmental and health risks [23]. In addition, photocatalysts can be employed for solar fuel generation, where sunlight is harnessed to drive reactions that produce value-added chemical products [24]. Another important application of photocatalysts is N_2_ fixation, a potential strategy for sustainable ammonia production, which plays a crucial role in both agriculture and industry [25]. Moreover, photocatalysis can enhance hydrogen production, positioning it as a promising fuel candidate for the electrification of industry and transportation [26,27]. Several original research studies on engineering design have been reported. For example, Zhang et al. synthesized pyrene-based covalent organic frameworks and tested them for H_2_O_2_ generation, achieving a rate of 2.961 mmol L^−1^ h^−1^ g^−1^ [28]. Meanwhile, RuO_2_/N,S-TiO_2_ photocatalysts exhibited 10.761 mmol L^−1^ h^−1^ g^−1^ of hydrogen with an apparent quantum yield of 10.0% in water containing glycerol [29]. Similarly, Shao et al. [30] investigated crystal-face regulation in porphyrin-based nanosheet photocatalysts for H_2_O_2_ production, observing that the exposed (400) surface achieved the highest rate of 29.33 mmol L^−1^ h^−1^ g^−1^, outperforming the (022) and (020) surfaces. In another study, conjugated microporous polymers were employed to develop a photocatalyst capable of producing hydrogen at a rate of 39.11 mmol L^−1^ h^−1^ g^−1^ under UV light in the presence of 1 wt.% Pt as cocatalysts [31].

This work aims to provide a comprehensive analysis of the primary engineering design strategies and their impact on enhancing the overall performance of photocatalytic materials. Although several reviews on photocatalytic materials have been published, most of them are extensive, covering multiple topics based on existing studies, yet they often lack comparative analysis from an engineering design perspective [32]. Some studies focus solely on a single design strategy, such as the single-atom photocatalysts approach [33], which may limit the understanding of beginners or practitioners in the field of photocatalysis. Thus, this study provides a comparative engineering perspective on multiple strategies and their impact on photocatalytic activity, stability, and charge transfer within an integrated framework.

## 2. Fundamentals of Photocatalysis

**Figure 1:**
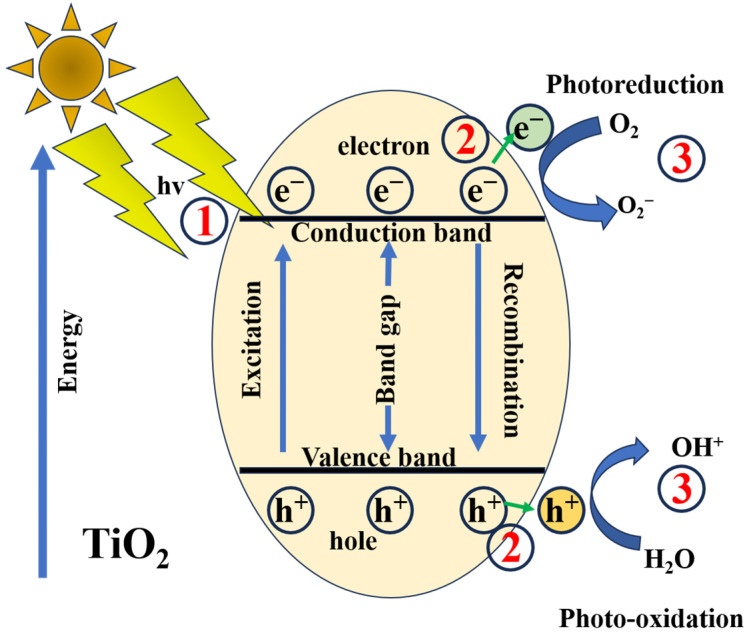
Schematic representation of the basic principle of photocatalysis. Figure designed and constructed based on [11,36,37].

Over millions of years of evolution, nature has refined highly efficient mechanisms, with photosynthesis serving as a prime example. In this process, CO_2_ is converted into carbon-based compounds with the concomitant release of O_2_, a transformation that can be mimicked using photocatalysts [34,35]. As reported, the photocatalytic process can be divided into three key stages: (i) light absorption, (ii) charge separation, and (iii) reaction mechanisms [33]. Figure 1 displays a schematic diagram of the photocatalysis principle in TiO_2_ nanoparticles, illustrating these three stages. When the material is excited by light of sufficient energy, electrons are promoted from the valence band to the conduction band, leaving behind holes in the valence band. These two bands are separated by a forbidden energy region known as the band gap. The photogenerated charge carriers (electrons and holes) subsequently migrate to the surface of the material, where they participate in photo-oxidation and photoreduction reactions.

The photocatalytic mechanism of TiO_2_ under light irradiation consists of a series of chemical reactions driven by photogenerated charge carriers [38], which are summarized as follows:

(1) $$\mathrm{Incident}\;\mathrm{energy}:\;TiO_{2}+hv\to e^{-}+h^{+}$$

(2) $$\mathrm{Reduction}:\;2h^{+}+2e^{-}\to H_{2};\;∆E=0\;V$$

(3) $$\mathrm{Oxidation}:\;2H_{2}O+4h^{+}\to O_{2}+4H^{+};\;∆E=1.23\;V$$

(4) $$\mathrm{Overall}:\;2H_{2}O\to 2H_{2}+O_{2};\;∆G=+237.2\;{\mathrm{kJmol}}^{-1}$$

TiO_2_, a semiconductor with a band gap of approximately 3.2 eV for the anatase phase and 3.0 eV for the rutile phase, initiates photocatalytic reactions when incident photons possess sufficient energy to excite electrons from the valence band to the conduction band, corresponding to the material’s band gap. Achieving water splitting additionally requires a thermodynamic potential of 1.23 V [39]. Engineering is critical in optimizing these processes, as it enables enhanced light absorption through morphological tuning and structural modifications, while the band gap itself can be engineered to maximize photocatalytic efficiency. In conventional semiconductive materials, the band gap ranges from 1.4 to 4.7 eV [40]. However, overall performance is also influenced by factors such as recombination, selectivity toward desired redox reactions, and long-term material stability. To address these challenges, strategies including doping, surface modification, and heterojunction construction have been implemented and are discussed in detail in the following sections.

## 3. Synthesis Approaches and Particle Growth Mechanisms

It has been extensively reported that the synthesis method plays a pivotal role in determining the final properties of nanostructures [41], as it allows control over particle growth and the achievement of desired morphologies through the adjustment of parameters such as temperature, pH, precursor concentration, and solvent type. Herein, we briefly discuss (i) hydrothermal synthesis, (ii) the sol-gel method, (iii) ball milling, (iv) solvothermal synthesis, and (v) the microwave-assisted hydrothermal process. Hydrothermal and solvothermal methods are the most commonly used approaches because they enable the formation of highly crystalline nanostructures with controlled morphology, such as nanorods, nanoplates, or nanocages, typically at temperatures between 120 and 200 °C [42]. The sealed autoclave environment promotes controlled nucleation and crystal growth, which is beneficial for generating materials with high surface area and abundant active sites for electrocatalysis. However, these techniques often require specialized high-pressure reactors and relatively long reaction times (several hours to days), which can increase operational costs and limit scalability [43]. An interesting study conducted by Ghamarpoor et al. comparing the main synthesis strategies revealed that the preparation route plays a decisive role in determining the structural and functional properties of ZnO/TiO_2_-based photocatalysts [44]. Their research concluded that hydrothermal synthesis produces highly crystalline nanostructures with well-defined morphologies such as nanorods, nanoflowers, and nanotubes, typically with particle sizes ranging from 10 to 50 nm. Similar findings were reported by Koozegar [45], who stated that hydrothermal synthesis of TiO_2_ promoted mixed crystalline phases, including both anatase and rutile, with slightly smaller crystallite sizes (~8–15 nm) compared to sol-gel (30–40 nm). Additionally, hydrothermal samples exhibit a significantly larger specific surface area of about 92 m^2^ g^−1^, while sol-gel materials have around 37 m^2^ g^−1^. Further comparative studies have highlighted that these structural differences strongly influence photoelectrocatalytic performance, particularly in terms of charge transport and recombination dynamics. For instance, several reports indicate that hydrothermally grown ZnO/TiO_2_ heterostructures exhibit superior photocurrent densities due to improved crystallinity and reduced defect-mediated recombination pathways [17].

Similarly, solvothermal synthesis provides excellent control over particle growth and morphology by tuning parameters such as solvent type, temperature, pressure, and precursor concentration, yielding well-defined nanoarchitectures with controlled crystallinity and exposed facets. For example, Rafiq et al. demonstrated that solvothermal growth of Ag_2_WO_4_/Sb_2_WO_6_ heterostructures in ethylene glycol significantly improved the charge transfer at the heterojunction interface, leading to enhanced photocatalytic degradation of organic pollutants compared to conventional hydrothermal samples [46]. Moreover, research on solvothermally synthesized ZnO and ZnFe_2_O_4_ nanoparticles demonstrates that uniform, nano-sized particulate structures (8–10 nm) produced by solvothermal routes show high photocatalytic degradation rates for dyes, highlighting the importance of controlled morphology and size distribution [47,48]. The main drawback of solvothermal synthesis is that it generally relies on organic solvents, such as ethanol or ethylene glycol, which can present environmental challenges and often require additional purification to remove residual solvents. In the case of the sol-gel method, it has been reported that it often produces more homogeneous thin films with good compositional control but tends to generate smaller pores and higher defect densities, which can act as charge recombination centers and limit photoelectrocatalytic efficiency [49]. Nevertheless, one drawback is that they frequently require post-synthesis thermal treatments (400–500 °C) to achieve adequate crystallinity, which increases energy consumption and may lead to particle agglomeration.

Other approaches, such as microwave-assisted hydrothermal synthesis, photodeposition, combustion, and sonochemical processes, can improve specific aspects of catalyst fabrication. For example, microwave-assisted methods significantly reduce synthesis time from hours to minutes [50], while photodeposition allows the selective incorporation of cocatalysts that improve electron transfer [51]. However, these techniques require specialized equipment, such as microwave reactors, irradiation sources, or ultrasonic systems, which increases the overall cost of implementation. Moreover, it is desired that synthesis methods employ deionized water as a solvent due to its low cost, low toxicity, and environmental compatibility. Even so, several methods, such as solvothermal synthesis, rely on organic solvents, including ethanol, acetone, methanol, or ethylene glycol, to control precursor solubility and particle growth [52]. The use of these solvents can introduce disadvantages, including higher material costs, solvent recovery requirements, environmental concerns, and additional purification steps. Finally, microwave-assisted hydrothermal synthesis offers significant advantages in terms of rapid heating and uniform nucleation, enabling the formation of smaller crystallites (around 10–15 nm) with narrow size distributions and improved crystallinity, ultimately leading to enhanced photocatalytic activity [53].

Once the advantages and disadvantages of the synthesis methods were discussed, the following lines focus on specific aspects highlighted by several authors, including procedure, temperature, chemical reagents, and other critical parameters. As mentioned above, the hydrothermal process is widely employed for the synthesis of nanomaterials due to its simplicity, scalability, and ability to produce well-defined nanostructures. For instance, Ortega et al. synthesized Eu-doped ceria with various morphologies, including nanoparticles, nanorods, nanocubes, and nanopolyhedra using a microwave-assisted hydrothermal method [54]. Morphology was controlled by varying only NaOH concentration and heating time. Nanorods were obtained using a 6 mol L^−1^ NaOH solution with a hydrothermal treatment of 8 min at 140 °C, whereas nanopolyhedrons were produced under the same duration at 180 °C. Characterization revealed that both morphology and structural parameters, such as lattice constants and defects, were affected, ultimately influencing the degradation efficiency of rhodamine B (RhB). The same synthesis approach was used to prepare bismuth tungstate (Bi_2_WO_6_) nanoplates by heating the precursor mixture at 180 °C for 15 h [55]. The reagents employed were 1 mmol L^−1^ sodium tungstate (Na_2_WO_4_) and 2 mmol L^−1^ of bismuth nitrate (Bi(NO_3_)_3_), dissolved in deionized water and HNO_3_, respectively, with the pH subsequently adjusted using NaOH solution. The resulting photocatalysts exhibited degradation efficiencies of 91.9% for RhB in 40 min and 87.2% for methyl orange (MO) in 210 min. As observed in the aforementioned examples, NaOH is commonly employed to adjust the pH and supply OH^−^ ions that promote hydrolysis and nucleation of metal precursors during hydrothermal synthesis. Consequently, the OH^−^ concentration strongly influences the nucleation rate and particle size: high alkalinity generally promotes rapid nucleation and the formation of smaller crystallites, whereas lower alkalinity favors slower growth and larger particles [56].

Solvothermal synthesis has also been applied to fabricate advanced nanostructures. For instance, tungsten trioxide (WO_3_) has been successfully obtained in both two-dimensional (2D) and 3D forms [57]. Morphological modulation of the 2D structures was obtained via hydrothermal synthesis assisted by the addition of oxalate or citric acid, whereas 3D structures were formed using a mixed solvent system of acetone and deionized water at a 2:5 volume ratio. Notably, decreasing particle size has been reported to shorten the diffusion length of photo-excited electrons and holes, consequently suppressing electron-hole recombination. Fe_3_O_4_ was successfully synthesized by Xu et al., who employed different urea concentrations as a dissolving agent in solvents such as ethylene glycol, water, and methanol [58]. The synthesis was carried out at 200 °C for reaction times ranging from 3 to 48 h. Similar conditions were employed to obtain CoFe_2_O_4_/Carbon nano composites, where glucose was dissolved in 35 mL benzyl alcohol, then 1 mmol cobalt acetate tetrahydrate and 2 mmol iron acetylacetonate were added and stirred for 2 h, and then heated at 200 °C for 24 h [59]. More notably, Bikerchalen et al. optimized the solvothermal synthesis of Bi_24_O_31_Cl_10_ in ethylene glycol by adjusting temperature, time, and pH to enhance photocatalytic performance [60]. They stated that the material synthesized at 160 °C for a period of 12 h exhibited optimal activity, achieving 99.98% degradation of Rhodamine B under visible light for 50 min. An interesting observation is that, in solvothermal synthesis, commonly used additives such as glucose, urea, and citric acid can significantly influence nucleation and growth rates, promote uniform morphology, and enhance both crystallinity and surface area.

On the other side, TiO_2_ was successfully synthesized via the sol-gel method by mixing deionized water, ethanol, and acetic acid. To control the morphology, sodium dodecyl sulfate (SDS), cetyltrimethylammonium bromide (CTAB), and polyethylene glycol (PEG) were added to the solvent, followed by the dropwise addition of 3.28 mmol L^−1^ titanium isopropoxide (Ti(OCH(CH_3_)_2_)_4_). The mixture was initially stirred at 25 °C for 24 h, and then the temperature was increased to 60 °C for an additional 24 h. The final step involves thermal treatment at 450 °C for 4 h. Characterization results revealed that anatase was the sole crystalline phase present, and the morphology strongly depended on the surfactant used. For instance, CTAB produces a worm-like morphology, whereas SDS yields spherical particles. Ortega et al. conducted a comprehensive study on the influence of the synthesis route on the performance of CoFe_2_O_4_ photocatalysts for hydrogen production, employing both chemical coprecipitation and ball milling techniques [61]. Briefly, in the coprecipitation method, a solution of Fe(NO_3_)_3_·9H_2_O and Co(NO_3_)_2_·6H_2_O was mixed with NaOH, and the recovered sample was thermally treated at 250 °C for 6 h, followed by 350 °C for 1 h. For mechanical milling, metallic Fe and Co_3_O_4_ were mixed in a molar ratio of 2:1, treated at 700 °C for 4 h, and subsequently subjected to mechanical milling for 12 h. X-ray diffraction (XRD) analysis revealed that the main crystalline phase in both materials was the CoFe_2_O_4_ spinel; however, the coprecipitation sample also contained a secondary Fe_2_O_3_ phase. Interestingly, the crystal size of the ball-milled sample was approximately 5 nm, about four times smaller than that obtained by coprecipitation. This reduction in crystal size, along with particle modifications, affected the band gap, which was 1.38 eV for the coprecipitation sample and 1.15 eV for the ball-milled sample, improving the H_2_ generation in the ball-milled sample, leading to nearly a fourfold enhancement in H_2_ generation for the latter. Ag-TiO_2_ provides another example in which the effect of the synthesis method was investigated by comparing photodeposition and formaldehyde-assisted microwave synthesis [62]. In this study, photodeposition exhibited superior photocatalytic performance; however, no significant differences were observed in terms of band gap or morphology, raising questions regarding which primary parameters are responsible for the improved activity. From the reviewed literature, it is evident that the hydrothermal method is the most commonly adopted synthesis approach due to its simplicity and cost-effectiveness. Table 1 provides a concise overview of selected synthesis methods, highlighting key factors such as solvent and capping agent, along with the resulting morphology and potential applications. It can be concluded that different preparation strategies and experimental parameters influence the structural characteristics of the obtained nanomaterials. In general, hydrothermal and solution-based methods are the most commonly employed approaches, typically using deionized water as the main solvent. Moreover, the synthesis conditions and the presence of additives such as NaOH, surfactants, or organic agents play a critical role in controlling the growth process, leading to a wide range of nanostructures, including nanorods, nanocubes, nanoplates, nanospheres, and irregular polyhedral particles.

On the other hand, capping agents are commonly employed during the synthesis of nanomaterials to control particle size and morphology, as they selectively adsorb onto specific crystal facets and thereby regulate crystal growth. A comprehensive and influential study was reported in 2017 by Phan et al., who proposed and validated the so-called penetrability model, which establishes a correlation between surfactant activity and particle growth [72]. To evaluate this model, a series of iron oxide nanoparticles was synthesized using eight cationic surfactants with an identical head group but varying hydrocarbon chain lengths, denoted as C*_n_*H_2*n*+2_(CH_3_)_3_NBr (*n* = 6, 8, 10, 12, 13, 14, 16, 18). Notably, the particle size decreased with increasing surfactant chain length, indicating that longer hydrocarbon tails provided stronger steric stabilization. Five years later, Ma and coworkers conducted a detailed study on the adsorption of three cationic surfactants with varying alkyl chain lengths on nanoparticles [73]. Zeta potential measurements were used to monitor changes in the isoelectric point, revealing that longer alkyl chains required lower surfactant concentrations to achieve similar effects. These results corroborate the observations reported by Phan et al., who also noted that surfactant behavior depends strongly on chain length.

**Figure 2:**
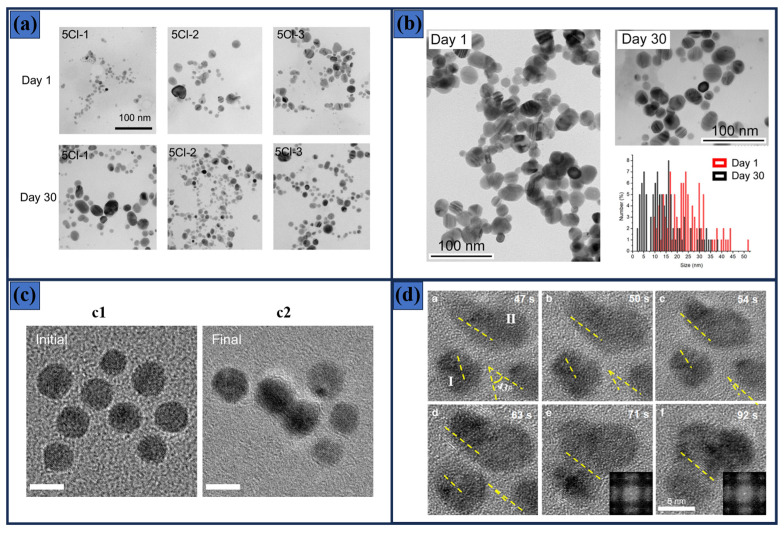
(**a**) TEM image for Ag nanoparticles synthesized in the presence of 5-chlorosalicylic acid with concentrations of 3 × 10^−3^ mol L^−1^, 1 × 10^−3^ mol L^−1^, and 1 × 10^−4^ mol L^−1^. (**b**) TEM image and particle size analysis for the as-synthesized AgNPs on the first day and after 30 days. Reproduced under the terms and conditions of the Creative Commons Attribution (CC BY) license (https://creativecommons.org/licenses/by/4.0/, accessed on 9 December 2025) [77]. (**c**) TEM image acquired before (**c1**) and after (**c2**) laser irradiation (4 mW or 7 mJ cm^−2^ per pulse for 7.5 min); the electron beam was off. Scale bars: 5 nm. Reproduced under the terms of The Journal of Physical Chemistry, Copyright © 2023, American Chemical Society [81]. (**d**) TEM image for ZnO particles showing the oriented attachment mechanisms. The yellow line shows particle rotation before contact. Reproduced under the terms of Creative Commons CC BY. Copyright © 2020 (http://creativecommons.org/licenses/by/4.0/, accessed on 12 December 2025) [82].

Beyond size control, surfactants can also influence particle size and morphology, as demonstrated in the synthesis of ZnS, where mercaptopropionic acid (MPA) and polyvinylpyrrolidone (PVP) yielded rod-like and spherical structures, respectively [74]. Although the underlying mechanism was not explicitly discussed in that study, it is well established that facet-selective adsorption plays a critical role as capping agents preferentially bind to specific crystal facets, thereby modifying their surface free energies and growth rates. Facets stabilized by capping agents grow more slowly and thus become more prominent in the final nanocrystal morphology. At elevated temperatures, thermodynamic control dominates, leading to the formation of equilibrium crystal shapes, whereas at lower temperatures, kinetic control prevails, often resulting in metastable morphologies [75]. Accordingly, the formation of distinct nanoparticle morphologies during synthesis is governed by a complex interplay of thermodynamic, kinetic, and chemical factors. Based on the literature, key particle growth mechanisms include Ostwald ripening, oriented attachment, and surface energy modulation by capping agents [76,77,78]. Ostwald ripening is a thermodynamically driven process occurring during nanoparticle growth, wherein smaller particles dissolve and redeposit onto larger ones, leading to an increase in average particle size. This phenomenon is closely related to the size-dependent solubility of spherical particles with radius *r*, which can be described by the Gibbs-Thomson equation [79,80]:

(5) $$C_{r}=C_{b}e^{\frac{2{\gamma V}_{m}}{rk_{B}T}}$$

where *Cr* is the solubility as a function of *r*, T is the temperature, γ is the surface energy, *V_m_* is the molar volume, and *k_B_* is the Boltzmann constant. The equation indicates that smaller nanoparticles exhibit higher solubility due to their larger surface-to-volume ratio, which increases their chemical potential. Figure 2a shows this effect, as observed by Alarcon et al. in freshly prepared Ag nanoparticle samples compared to those aged for 30 days. A clear increase in particle size was observed, which was attributed to the Ostwald ripening phenomenon [77].

In this regard, Ireneusz et al. investigated the photochemical growth of Ag nanoparticles on TiO_2_ coatings and found that the UV illumination time plays a critical role in determining particle size [83]. For instance, a short exposure of 20 s led to the formation of small nanoparticles, as confirmed by scanning electron microscopy (SEM), whereas extending the illumination by only 10 s resulted in particle coarsening and the formation of large particles. A similar trend was reported in ref. [77], where the authors monitored particle size evolution in freshly synthesized samples and after 30 days of aging, as depicted in Figure 2b. In this case, released Ag species interacted with existing nanoparticles, promoting their growth into larger structures, in accordance with the Ostwald ripening mechanism. Direct experimental evidence of this phenomenon was reported in 2023 by Alcorn et al. [81], who used a transmission electron microscope (TEM) equipped with a laser to excite a Au-Cu alloy sample. The plasmonic excitation favored Ostwald ripening, as illustrated in Figure 2c, where nanoparticles were observed to coalesce and form agglomerates. Oriented attachment (OA) is another process commonly observed in crystal growth, in which crystalline particles align their atomic lattices and merge along matching crystal planes to form larger, single-crystalline structures [84]. This process is energetically favorable as it reduces the overall surface energy of the system by eliminating high-energy interfaces, thereby stabilizing the growing crystal. In polar semiconductors such as ZnO, the mechanism is strongly influenced by electrostatic interactions associated with the polar (0001) facets, which create dipole moments capable of generating attractive torques between particles. Liu et al. conducted a real-time investigation using in situ TEM to observe this effect in ZnO nanoparticles, revealing that forces and torques arising from a combination of electrostatic and dipolar interactions govern particle behavior at the 5 nm scale [82]. The former is illustrated in Figure 2d, where individual ZnO nanoparticles dispersed in methanol containing 1 mM Zn^2+^ rotate to align their crystallographic axes (as observed in subfigures a–f) and eventually merge to form a single crystal after 92 s. Several representative studies have further expanded the understanding of this mechanism. Oriented attachment in semiconductor nanocrystals can occur through intermediate metastable assemblies, where particles first form loosely ordered aggregates before undergoing crystallographic fusion [84]. Also, OA contributes to the formation of ZnO nanorods and nanowires, particularly under hydrothermal conditions where the polar nature of ZnO surfaces favors directional growth along the c-axis [85].

## 4. Morphological and Structural Design

The influence of morphology on structural features of photocatalytic activity is well established and has been extensively explored across a wide range of materials [86]. Early work by Hu and co-workers demonstrated that MoS_2_ morphology and size critically affect visible light, the photocatalytic degradation of methyl orange (MO), with nanoslices (41 m^2^ g^−1^) outperforming nanoballs (19 m^2^ g^−1^) and bulk structures (6 m^2^ g^−1^) [87] and achieving nearly 90% decolorization of MO within 150 min, an effect attributed to quantum-confinement-induced-band-gap-modulation. In this sense, Cen et al. conducted an in-depth study on the morphology (microspheres and non-uniform nanoparticles) and crystallinity of (BiO)_2_CO_3_ during nitric oxide (NO) degradation under UV irradiation [88]. The microspheres exhibited a photocatalytic efficiency of 42.6%, whereas the nanoparticles achieved only 24.8%. This behavior was attributed primarily to differences in surface area and pore-size distribution. Specifically, the microspheres possessed a relatively large surface area of 34.5 m^2^ g^−1^, while the nanoparticle-based material showed minimal interaction with N_2_, indicating a lack of accessible porosity.

**Figure 3:**
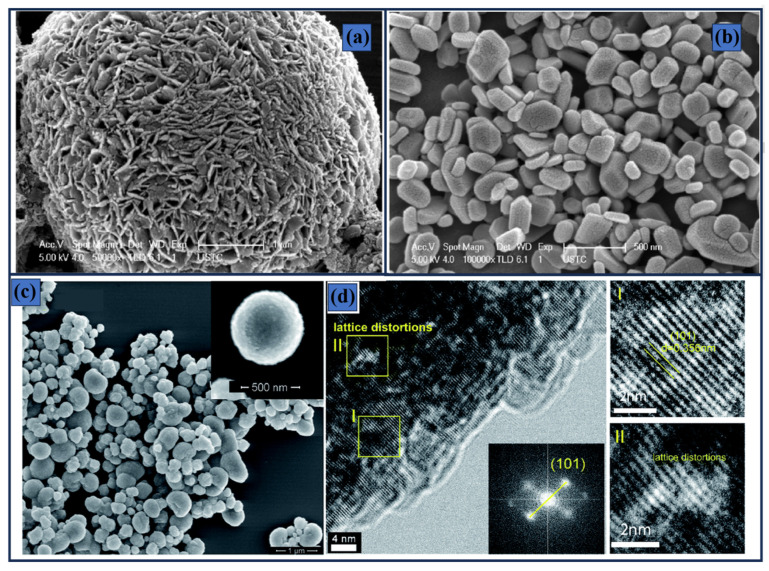
(**a**,**b**) SEM micrographs for (BiO)_2_CO_3_. (**a**) Microspheres obtained by using a low sodium carbonate concentration. (**b**) Non-uniform nanoparticles obtained by high sodium concentration in the hydrothermal synthesis. Reproduced under the terms of Industrial & Engineering Chemistry Research. Copyright © 2014, American Chemical Society [88]. (**c**) SEM image of TiO_2_ microspheres showing lattice distortion and defects. (**d**) HRTEM image highlighting lattice distortion, with FFT of area I and enlarged views of regions I and II. Reproduced under the terms of Creative Commons Attribution-NonCommercial 3.0 [90].

Notably, crystallinity was also identified as a critical factor, as the most efficient photocatalysts exhibited lower crystallinity, which promoted a red shift in absorption edge and resulted in a reduced band gap of 3.14 eV. Figure 3a,b shows the drastic changes in the morphology of (BiO)_2_CO_3_, revealing that a small sodium carbonate concentration leads to highly porous microspheres. This structural feature enhances reactant diffusion and promotes multiple light scattering, thereby improving light-harvesting efficiency. It is noteworthy that the NO degradation efficiency of (BiO)_2_CO_3_ remains relatively low compared with other photoelectrocatalysts, such as the optimized δ-MnO_2_ reported by Li et al., reaching removal efficiencies of up to 80% [89]. In that study, three MnO_2_ phases (δ-MnO_2_, α-MnO_2_, and γ-MnO_2_) were systematically investigated, exhibiting self-assembled folded nanosheets, rod-like structures, and urchin-like nanospheres, respectively. Detailed analysis revealed that structural parameters played a decisive role in determining photocatalytic performance. The TEM images confirmed the presence of the (001), (002), and (110) facets, influencing electron distribution along these planes, thus promoting the separation of photogenerated charge carriers. Moreover, lattice distortion was preferentially observed in δ-MnO_2_, indicating the formation of oxygen vacancies, a finding further supported by X-ray photoelectron spectroscopy (XPS) analysis. These features resulted in both a reduced band gap (1.38 eV) and a lower charge-transferresistance (R_ct_). Along similar lines, Xu et al. [90] reported that the introduction of Ti^3+^ defects into TiO_2_ microspheres induces pronounced lattice distortion, as confirmed by the TEM and high-resolution TEM (HRTEM) images shown in Figure 3c,d. The presence of these defects significantly enhances the photocatalytic activity of the material toward RhB degradation, achieving efficiencies of up to 75%. Furthermore, the photocurrent response increased dramatically from 1.78 to 53.78 μA cm^−2^, which was attributed to the suppression of electron and hole recombination. UV-vis diffuse reflectance spectroscopy revealed that the valence band maximum of pristine TiO_2_ lies 3.1 eV below the Fermi level, whereas the Ti^3+^-modified sample exhibited an upward shift of about 0.36 eV, indicating a noticeable modification of its structure.

As mentioned above, controlling the morphology of photocatalysts is an effective strategy for enhancing photoactivity, as it can increase the surface area and create additional active sites. However, Cheng et al. reported that the highest-performing TiO_2_ material was not directly correlated with surface area but instead depended on the anatase-to-rutile phase ratio, with a 70:30 composition yielding superior photocatalytic performance [91]. These findings expose the complexity of the mechanisms governing photocatalytic activity and underscore the need for careful consideration when tailoring material properties. To further elucidate the influence of morphology on photocatalytic performance, particular attention is given to the study by Roškarič et al. [92], who compared three TiO_2_ morphologies: anatase nanoparticles (TPs), poorly crystalline anatase nanotubes (aTTs), and well-crystalline anatase nanorods (TRs), all comprising exclusively the anatase phase, for the degradation of bisphenol. By eliminating the phase effect, this study enabled a focused evaluation of morphology alone. Poorly crystalline nanotubes were prepared via hydrothermal treatment of commercial TiO_2_ (DT-51) using 10 mol L^−1^ NaOH, while nanorods were subsequently obtained by thermally treating the hydrothermally obtained sample at 500 °C for 2 h. Finally, the resulting samples were mixed with g-C_3_N_4_ in a 1:1 ratio using a mortar to fabricate the photocatalysts.

**Figure 4:**
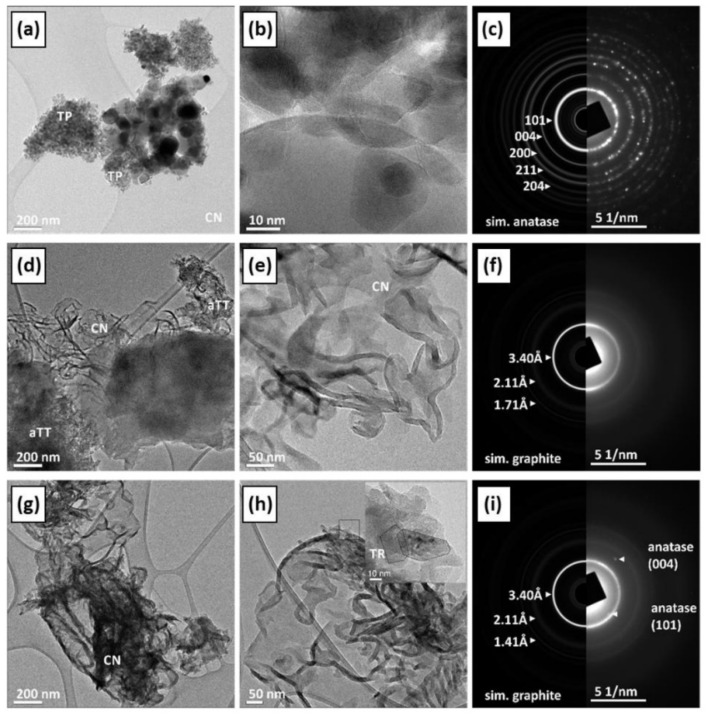
TEM pictures of the g-C_3_N_4_/TiO_2_ photocatalysts. (**a**–**c**) g-C_3_N_4_ with TiO_2_ nanoparticles; (**d**–**f**) g-C_3_N_4_ with poorly crystalline TiO_2_; and (**g**–**i**) g-C_3_N_4_ with TiO_2_ nanorods. Reproduced under the terms and conditions of the Creative Commons Attribution (CC BY) license (https://creativecommons.org/licenses/by/4.0/) [92].

Figure 4 shows the TEM images of the synthesized materials, revealing pronounced morphological differences induced by thermal treatment. It is noteworthy that the authors reported nearly identical carbon and nitrogen contents across all samples. Therefore, the observed variations in photocatalytic activity can be attributed primarily to morphological effects. The bisphenol A degradation results show that the nanoparticle-based photocatalyst exhibits the lowest degradation efficiency (11.8%), whereas the nanorod-based material achieves the highest degradation efficiency (26%). These results are consistent with the electrochemical impedance spectroscopy (EIS), in which the nanorods exhibit the lowest R_ct_ value (0.67 MΩ), thereby confirming the crucial role of morphology in governing photocatalytic performance following thermal treatment.

The incorporation of graphitic carbon nitride (g-C_3_N_4_) into the TiO_2_ matrix and other conventional photocatalysts has been widely adopted as an effective engineering design strategy. Deng et al. investigated the photocatalytic degradation of methylene blue (MB) using spherical, spindle-shaped, and cubic TiO_2_ nanoparticles supported on g-C_3_N_4_ [93]. Spherical TiO_2_ nanoparticles were synthesized via a hydrothermal process, whereas spindle- and cubic-shaped nanoparticles were obtained through the use of ethylenediamine and sodium oleate as shape-directing agents, respectively. From an engineering perspective, morphological control originates from the selective adsorption of these directing molecules during the nucleation and growth stage. Specifically, ethylenediamine preferentially adsorbs onto crystal planes parallel to the c-axis, promoting spindle-shaped TiO_2_, while sodium oleate selectively binds to the (001) and (100) facets, leading to the formation of cubic structures.

The described synthesis procedure exclusively favored the formation of the anatase phase, as confirmed by XRD analysis. The reported BET surface areas were measured to be 150, 86, and 99 m^2^ g^−1^ for spherical, spindle-shaped, and cubic TiO_2_, respectively. These materials were further mixed with g-C_3_N_4_ and used as a photocatalyst for methylene blue (MB) degradation. A key finding was that the cubic TiO_2_ structure exhibited lower photoluminescence (PL) intensity, indicating suppressed charge carrier recombination. Correspondingly, this photocatalyst achieved the highest MB degradation activity with a rate of 13.3 × 10^−2^ h^−1^, despite not possessing a large surface area. To gain further insight, a detailed examination of the XRD patterns confirmed anatase as the sole crystalline phase in all samples. Notably, the cubic TiO_2_ structure exhibited superior crystallinity, as evidenced by the sharper and more defined (101) and (100) diffraction peaks compared with other morphologies. Furthermore, the authors reported that this material forms face-to-face (2D-2D) interfacial contact with g-C_3_N_4_, which is more favorable for efficient charge carrier migration.

It can be inferred that both morphology and structural characteristics significantly influence the efficiency of electrocatalysts, making it challenging to discern which factor is dominant. In this context, particular attention should be given to the study by Yin et al., who investigated this issue in detail [94]. Bi_3_Fe_0.5_Nb_1.5_O_9_ (BFNO) was synthesized by using both hydrothermal (BFNO-H) and solid-state (BFNO-S) methods, resulting in hierarchical and disordered micron-scale morphologies, respectively. Initially, the superior performance of BFNO-H was attributed to its larger specific surface area. However, even after normalizing the results to account for surface area, BFNO-H continued to outperform BFNO-S. XRD and TEM analyses indicated the presence of exposed (001) and (110) facets, which are conducive to efficient generation and transport of charge carriers.

## 5. Nanostructures Across Dimensions

### 5.1. Quantum Dots and 1D Nanostructures

Quantum dots and one-dimensional (1D) nanowires have been explored in photocatalysts [95]. Nonetheless, they are typically integrated with 2D or 3D structures rather than used independently [96]. Quantum dots generally require a supporting matrix to prevent agglomeration and to ensure efficient charge separation due to their extremely small size and high surface energy. For example, γ-Fe_2_O_3_ quantum dots were incorporated into a metal–organic framework-801 (MOF-801) porous matrix using a double-solvent method combined with an in situ reduction approach [97]. The resulting composite exhibited a degradation efficiency of 84.15% for Acid Orange 7 after 180 min of visible light irradiation, which was attributed to the reduced band gap of 3.1 eV, compared to 4.4 eV for pristine MOF-801. Similar behavior has been reported for other MOF-based composites in which semiconductor nanoparticles or quantum dots are embedded inside the porous framework. For instance, ZnO quantum dots confined within MOF-801 prepared by a double-solvent method exhibited a 2.35-fold increase in photocatalytic degradation rate compared with pristine MOF-801 due to improved charge separation and quantum confinement effects [98]. Likewise, MnO_2_-modified MOF-801 composites displayed enhanced visible-light photocatalytic activity as a result of band-gap narrowing and increased active surface area [99]. Other complex materials used carbon quantum dots (CQDs) deposited on BiVO_4_ to obtain an S-scheme heterojunction capable of absorbing visible light up to 750 nm [100]. This heterostructure also exhibited piezo-photocatalytic activity, showing efficient degradation of antibiotics. XRD analysis exposed that the incorporation of CQDs did not affect the crystallinity of BiVO_4_. No distinct diffraction peaks corresponding to CQDs were detected, owing to their amorphous nature. Nevertheless, their presence was verified by energy-dispersive X-ray spectroscopy (EDS) elemental mapping. Tetracycline (TC) degradation under visible light irradiation coupled with ultrasonic treatment exhibited a rate of 0.0517 min^−1^. In a related study, Zhao et al. [101] employed N-doped carbon quantum dots (N-CQDs) and graphitic carbon nitride quantum dots (CNQDs) to modify TiO_2_ photocatalysts via a hydrothermal synthesis, using ammonium citrate as a precursor in the presence and absence of urea. XPS revealed that both samples contained nitrogen species, which significantly enhanced photocatalytic activity. Notably, the C-N peak in the CNQDs sample accounted for 18.9%, compared with 16.5% in the CQDs sample. The study also highlighted that excessive quantum dot loading could reduce photocatalytic activity, as they competed with TiO_2_ for photon absorption, limiting the generation of photogenerated charge carriers and reducing charge transfer efficiency.

To further evaluate the effect of the quantum dot loading, the photocatalytic performance of Zn_0.5_Cd_0.5_S (ZCS) quantum dots deposited onto a flower-like BiOI was examined [102]. Incremental loading from 1% to 3% elicited a noticeable decline in photoactivity, indicating that 1% constitutes the optimal loading. At this concentration, the degradation rate of RhB reached 99.2% upon 75 min of irradiation. The reduction in activity at higher loadings is primarily ascribed to the intrinsically low photocatalytic efficiency of ZCS and has a deleterious effect on the crystallinity. In a similar context, Malitha et al. reported that augmenting the content of CQDs and ammonium persulfate in cobalt-zinc ferrite resulted in only a significant improvement in the degradation rate of Reactive Yellow 145, a phenomenon attributed to the concomitant reduction in the accessible active surface area of the ferrite [103]. A particularly effective strategy involved the use of CdS quantum dots to modify TiO_2_-anatase/silica core–shell nanostructures [104]. The resulting composite exhibited uniformly dispersed particles with an average diameter of approximately 1.8 nm and demonstrated remarkable photocatalytic activity, achieving 91% degradation of MB under UV irradiation within 240 min, corresponding to an apparent rate constant of 0.01 min^−1^. SEM and TEM analyses confirmed a dense and well-defined morphology. The authors reported that anatase nanoparticles nucleated and grew around the CdS quantum dots via the Ostwald ripening mechanism, while the hydrophobic tails of CTAB facilitated the formation of a SiO_2_ shell surrounding the composite. The synergetic interaction among the components resulted in a reduced band gap of 3.2 eV compared with 3.9 eV for the corresponding material without the silica core. Considering the works discussed above, it can be inferred that QDs can significantly enhance photocatalytic performance due to their quantum confinement effect, tunable band structure, and strong light absorption, which promote visible-light harvesting and improve charge separation in semiconductor heterostructures. However, their benefits strongly depend on controlled loading, since excessive QD content may compete for photon absorption, reduce charge transfer efficiency, and decrease active surface area, ultimately lowering photocatalytic performance.

In contrast, 1D nanostructures such as nanowires or nanotubes are frequently employed as scaffolds or electron transport pathways, providing mechanical stability and facilitating directional charge transfer when coupled with higher-dimensional architectures. Jawale et al. [105] reported the synthesis of TiO_2_ nanowires with diameters ranging from 7 to 10 nm, which evolved from spherical-wire-flake-like morphologies depending on the reaction time and temperature. The optimized nanowires exhibited outstanding photocatalytic performance, achieving 100% MB degradation within 30 min, with an apparent rate constant K_app_ = 13.54 × 10^−2^ min^−1^. This study emphasizes the importance of controlling synthesis conditions, as non-optimal parameters resulted in the formation of spherical nanoparticles or nanorods instead of nanowires. As mentioned above, TiO_2_ nanowires are typically not used alone but often combined with other elements to enhance their performance. For example, Giuffrida et al. doped TiO_2_ nanowires with Fe, obtaining a rutile phase, a noteworthy result, since most synthesis methods preferentially yield the anatase phase [106]. The incorporation of Fe via ion implantation improved film conductivity and tuned the band gap, as confirmed by the reduction from 3.2 eV in undoped TiO_2_ to 2.8 eV in the Fe-doped sample. More complex heterostructures have also been developed, such as CdS/g-C_3_N_4_ composite modified with Ni(OH_2_), where this was tested as a photocatalyst for H_2_ generation under visible light irradiation [107]. This material delivered a hydrogen generation rate of 115.18 μmol h^−1^ mg^−1^, which is approximately 26 times higher than that of the unmodified composite. Indeed, the distinction between nanowires and nanorods is often debated, with a practical criterion being the apparent aspect ratio, defined as the relationship between length and diameter (L/D), which typically exceeds 30 for structures to be classified as a nanowire [108].

**Figure 5:**
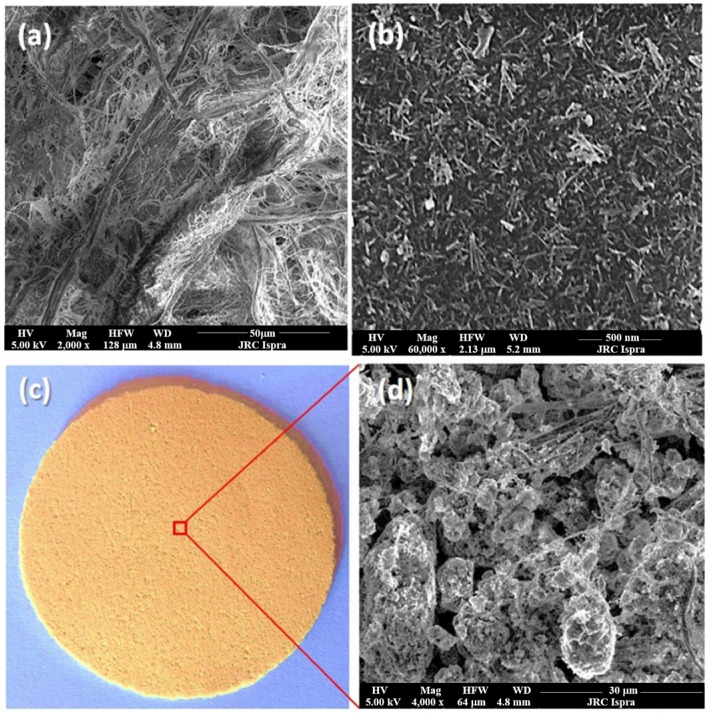
TEM images for the TiO_2_ NW@Fe_2_O_3_ hybrid membrane. (**a**) Cellulose; (**b**) TiO_2_ nanowire TiO_2_; and (**c**,**d**) hybrid membrane containing TiO_2_ NW@Fe_2_O_3_. Reproduced under the terms and conditions of the Creative Commons Attribution (CC BY) license (https://creativecommons.org/licenses/by/4.0/) [109].

Hybrid structures have attracted considerable attention due to their potential to yield cost-effective materials. In this context, TiO_2_ nanowires were incorporated into membranes and evaluated for the degradation of MB under UV irradiation [109]. Commercial TiO_2_ was first transformed into nanowires via a solvothermal process, then washed with HCl and calcined at 500 °C, with the resulting morphology presented in Figure 5b. The obtained nanowires were then dispersed in an aqueous FeCl_3_·6H_2_O solution, transferred to an autoclave, and treated at 90 °C for 9 h. The product was subsequently washed with 0.1 M NaOH, dried at 50 °C for 12 h, and calcined at 500 °C for 2 h to yield TiO_2_ nanowires containing 5 wt% Fe_2_O_3_. The composite membrane was prepared by blending TiO_2_ NW@Fe_2_O_3_ with cellulose, followed by filtration and drying at 40 °C, see Figure 5c. Impressively, the membrane achieved 90% degradation of MB after 60 min of irradiation.

### 5.2. Two-Dimensional (2D) Nanostructures

Two-dimensional semiconductors such as nanosheets with large porosity have been effective photocatalysts due to enhanced charge transfer, efficient mass transport, abundant active sites, and improved light harvesting [110]. In this context, ZnV_2_O_6_ nanosheets were synthesized via a solvothermal method and evaluated for photocatalytic degradation of RhB [111]. A Central Composite Design (CCD) approach was adopted, considering pollutant dose, pH, photocatalyst loading, and irradiation time, to optimize a material capable of achieving 97% degradation efficiency. Although morphological and structural features were not explicitly considered in the CCD analysis, two distinct morphologies were observed: trapezoidal-shaped structures, corresponding to the monoclinic ZnV_2_O_6_ phase, and spherical particles associated with the monoclinic Zn_2_V_2_O_7_ phase. Morphology tuning of nanosheets was further addressed by Linh et al., who employed thermal polymerization assisted by a urea precursor to control the morphology of g-C_3_N_4_ [112]. By varying the annealing temperature, stacked-layer, porous, and ultrathin morphologies were obtained, with the ultrathin g-C_3_N_4_ achieving complete degradation of a 10 ppm RhB solution within 60 min under solar irradiation. Textural analysis revealed that increasing annealing time led to broadening and enhanced intensity of the (200) reflection peak, indicating thermal exfoliation, reduced layer thickness along the c-axis, and improved interlayer stacking of the aromatic structure. Band gap values were also affected, with annealing durations of 0.5, 1.5, 2.5, and 3.5 h yielding band gaps of 2.63, 2.57, 2.55, and 2.52 eV, respectively. Similarly, ballmilling for 24 h in the presence of solvents such as isopropanol, ethanol, and deionized water induced significant morphological changes in g-C_3_N_4_, leading to laminar structures with reduced particle size and a decrease in XRD peak intensity due to disruption of the interlayer structure from van der Waals bond breakage [113]. Wang et al. observed that the two-dimensional layered structure of g-C_3_N_4_ modified with CdS facilitates efficient electron transmission, and the high specific surface area increases active sites [114]. These studies reveal the crucial role of morphology in photoactive materials, as it not only influences the available surface area but can also contribute to band-gap tuning and charge transfer behavior.

**Figure 6:**
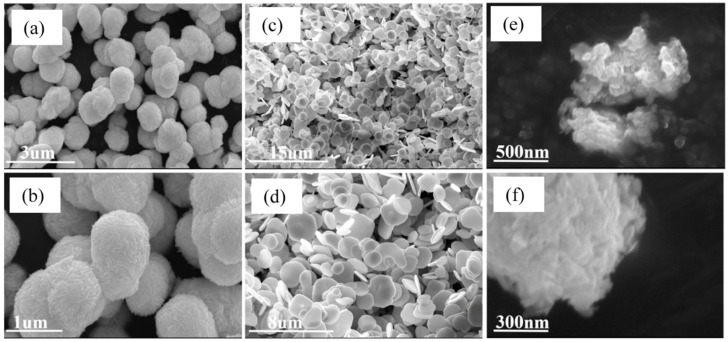
SEM micrographs of BiOCl samples at different magnifications: (**a**,**b**) BiOCl-1, (**c**,**d**) BiOCl-2, and (**e**,**f**) BiOCl-3. Reproduced under the Creative Commons CC-BY-NC license, © 2024 The Authors. Published by Elsevier B.V. [117].

A critical issue in designing a 2D photocatalyst is the potential mismatch between elements or compounds incorporated into the system. Traditional noble and non-noble metals often exhibit lattice mismatch with 2D covalent organic frameworks [115]. For instance, Prussian Blue analog nanosheets composed of PdTCNi/HCFe were synthesized via a co-precipitation method and employed for the photocatalytic degradation of crystal violet [116]. The XRD of PdTCNi/HCFe displayed defined reflections with an estimated crystallite size of 29.25 nm, which is evidence of lattice distortion. Morphological observations showed a plate-like structure at low magnification, while higher magnifications revealed small spherical structures linking the nanosheets. Optical characterization indicated a band gap of 2.96 eV, with strong light-matter interaction attributed to the nanosheet morphology, arising from the large surface area and high aspect ratio. A similar behavior was reported by Luo et al., who investigated the transformation of BiOCl nanosheets (shown in Figure 6) into a cotton-like morphology [117].

The BiOCl sample prepared via the solvothermal method exhibited predominantly spherical particles with diameters of about 1 µm. In contrast, BiOCl-2 (synthesized via a hydrothermal route without surfactant) showed a morphology consisting of thin platelets ranging from 1 to 3 µm in diameter. Meanwhile, BiOCl-3 (obtained through the hydrothermal process in the presence of PVP) consisted of ultrathin nanosheets that assemble into a cotton-like structure, with individual diameters estimated between 20 and 50 nm. This morphological feature enhanced the specific surface area, increasing the number of active sites and thereby improving the photocatalytic performance.

More recently, Chen et al. developed a platinum single-atom-decorated MoS_2_/ZnIn_2_S_4_ (PtSA-MoS_2_/ZnIn_2_S_4_) heterostructure to enhance photocatalytic activity [118]. In this system, the intimate coupling of the two-dimensional components forms a well-aligned 2D/2D heterojunction that induces favorable band-structure modulation in ZnIn_2_S_4_, maximizes interfacial contact, and enhances transfer separation. Moreover, the presence of highly active platinum single-atom cocatalysts further promoted the hydrogen evolution reaction (HER) by lowering the reaction overpotential and providing abundant active sites.

### 5.3. Three-Dimensional (3D) Nanostructures

ZnO nanoflowers with an average crystallite size of about 55 nm were obtained by the hydrothermal method assisted by oleic acid, achieving high crystalline purity [119]. The authors reported that the additive concentration played a key role in determining the flower-like morphology by directing the anisotropic growth process. Furthermore, variations in oleic acid concentration were found to alter the degree of supersaturation, which in turn influenced structural parameters such as crystallographic plane orientation. Oleic acid acts as a capping and structure-directing agent due to its carboxylate group, which can coordinate with Zn^2+^ ions or adsorb on specific ZnO crystal facets, thereby modifying surface energies and selectively inhibiting growth along particular crystallographic directions [120]. This selective adsorption promotes anisotropic growth, favoring the formation of hierarchical assemblies such as nanorods or nanosheets that subsequently aggregate into flower-like architectures. In ZnO, the polar (0001) plane possesses higher surface energy than non-polar facets, which naturally drives preferential growth along the c-axis [121]. However, surfactants such as oleic acid can partially block these surfaces and redirect crystal growth laterally, facilitating the radial arrangement of nanorods characteristic of nanoflowers [122].

On the other hand, g-C_3_N_4_ was engineered into tubular structures through methanesulfonic acid-induced thermal polymerization of melem, which introduced carbon vacancies and consequently enhanced its photocatalytic activity for H_2_ generation, reaching 4035.8 μmol g^−1^ h^−1^ [123]. This enhancement was primarily attributed to a significant increase in the specific surface area to 105.2 m^2^ g^−1^, providing a greater number of active sites for the reaction. SEM and TEM analyses revealed a hollow, nanosheet-stacked tubular morphology, composed of multiple nanosheet layers aligned along the tube axis. Notably, X-ray photoelectron spectroscopy (XPS) analysis showed that high-resolution C, N, and O spectra exhibited a 68.38% decrease in the C/N atomic ratio, which was attributed to the formation of carbon defects during thermal treatment. Another critical factor contributing to the improved H_2_ evolution performance is the formation of carbon vacancies during thermal treatment. These defects can introduce localized electronic states within the band structure of g-C_3_N_4_, which promote charge separation and increase the density of catalytically active sites. Carbon vacancies are also known to enhance the adsorption and activation of reactant molecules and facilitate electron transfer processes, thus improving photocatalytic reduction reactions [124]. Nevertheless, although defect engineering significantly enhances photocatalytic performance, it must be considered that excessive defect formation can introduce deep trap states that act as recombination centers, reducing charge carrier lifetime [125].

**Figure 7:**
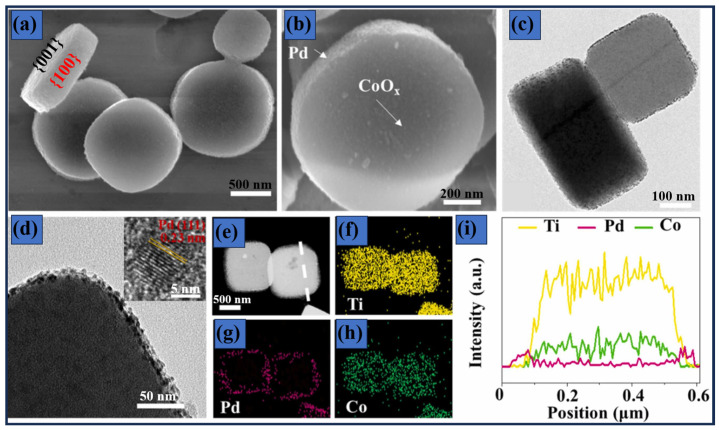
Characterization of the modified MOF support with Pd and CoO_x_. (**a**,**b**) SEM micrographs, (**c**) TEM image, (**d**) HRTEM image, (**e**–**h**) HAADF-STEM and corresponding element mapping images, and (**i**) line scan spectra of PMC. Reproduced under the term Creative Commons CC-BY, © 2024 The Author(s). Published by Elsevier B.V. [129].

**Figure 8:**
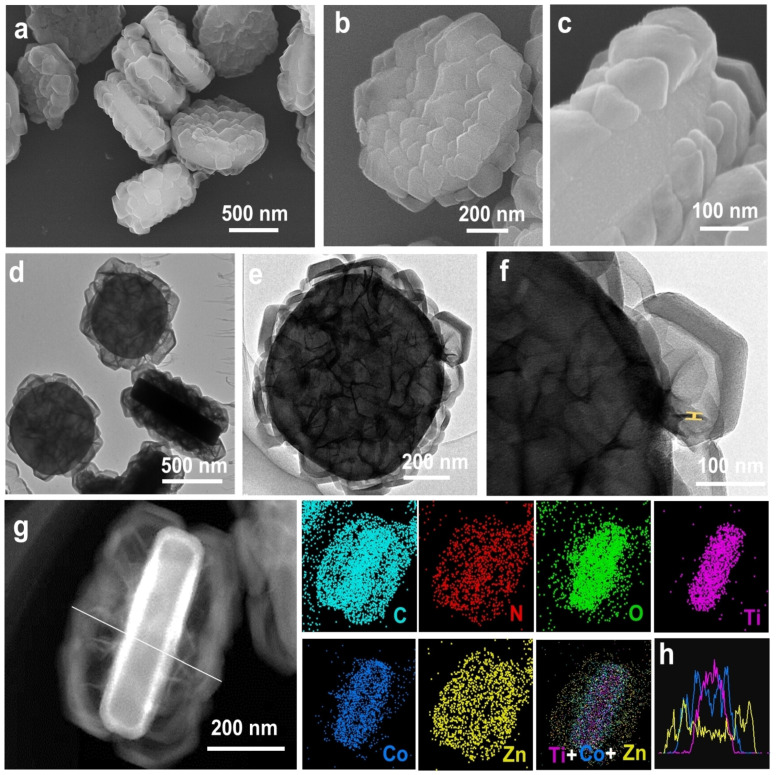
Morphological and chemical characterization of the NMCZ sample. (**a**–**c**) SEM micrographs. (**d**–**f**) TEM images. (**g**) HAADF-STEM image and corresponding elemental mapping. (**h**) Line scan profile of elemental distribution. © 2024 Wiley-VCH GmbH [163].

MOFs have been widely used as photocatalysts owing to their intrinsic semiconducting behavior, high specific surface area, tunable band gap, defect engineering capability, structural tunability, and other outstanding properties [126,127]. These features can be strategically tailored to enhance light harvesting, promote efficient charge separation, and suppress charge recombination, thereby improving photocatalytic activity [128]. Yuan et al. developed a Pd- and CoO_x_-based MOF photocatalyst for H_2_O_2_ production coupled with biomass oxidation [129]. A notable aspect of this work was the crystal facet-dependent spatial separation of the Pd and CoO_x_, where the (100) and (001) facets of the MOF promoted directional migration of photogenerated charge carriers. Facet engineering is a well-established strategy in photocatalytic materials research, as it enables a dual mechanism whereby photogenerated electrons accumulate on one facet while holes migrate to another [130]. In this study, Yuan and co-workers provided a detailed elucidation of the interaction between the cocatalyst and the MOF support, revealing that the preferential growth of Pd on the (100) facet and CoO_x_ on the (001) facet (see Figure 7) is mainly driven by charge effects. For example, the (100) facets expose Ti^4+^ species that exhibit a strong affinity toward PdCl_4_^2−^ anions, a finding further corroborated by the elemental mapping analysis shown in Figure 7e and Figure 8.

More importantly, control experiments using methanol instead of diethylene glycol revealed a pronounced solvent effect on nucleation. Methanol favored rapid homogeneous nucleation in solution rather than heterogeneous nucleation on the support, resulting in non-selective particle adhesion to the substrate. Although the original study did not explicitly address the role of solvent properties in governing nucleation kinetics, extensive literature suggests that parameters such as polarity, viscosity, ionic strength, protic versus aprotic character, redox chemistry, and hydrogen bonding interactions critically influence nucleation processes [131,132,133]. These solvent-induced effects not only drive significant morphological alterations in the Pd- and CoO_x_-modified MOF but also trigger structural modifications that modulate the photocatalytic activity. As a result, H_2_O_2_ production was markedly enhanced, reaching 74.8 mmol g^−1^h^−1^, which substantially exceeds previously reported values, including 1.676 mmol g^−1^h^−1^ for functionalized conductive MOFs reported by Choi et al. [134] and 10.4 mmol g^−1^h^−1^ for Pd-anchored MOF [135].

As discussed previously, certain crystal facets are more favorable for nanoparticle nucleation and growth. From an engineering perspective, the rational design of supports that expose multiple facets can significantly enhance photocatalyst efficiency. In this context, Du et al. reported the synthesis of MIL-125-NH_2_ polyhedrons (MIL = Materials Institute Lavoisier) with well-defined (001)/(111) facet exposures [136], which exhibited a remarkable H_2_O_2_ production rate of 128.6 mmol L^−1^ g^−1^ h^−1^ upon modification with Pd^0^ and PdO. Structural characterization revealed that Pd preferentially grows on the top surfaces of the polyhedra, while PdO predominantly deposits on the lateral facets. Collectively, findings demonstrate that facet engineering plays a vital role in enhancing photocatalyst performance by modulating the electronic structure, improving charge-carrier transport, and increasing the exposure of catalytically active sites [137,138]. Alternative strategies have been proposed in MOF-based photocatalysts to address issues related to degradation rate and material recovery. For instance, a Cu-based MOF integrated into an alginate substrate [139], which not only enhanced photocatalytic efficiency but also contributed to the development of environmentally friendly materials. This material demonstrated 99% decolorization of Congo Red (CR) and maintained a high performance of 90% even after ten consecutive cycles. Hemdan et al. reported an outstanding study in which sodium alginate was incorporated to fabricate a Cu-BTC@Alg/Fe_3_O_4_ composite. In this system, BTC (1,3,5-benzenetricarboxylic acid) served as the organic binder for the synthesis of the Cu-based MOF [140]. The composite exhibited a specific surface area of 160 m^2^ g^−1^ and an adsorption capacity of 200 mg g^−1^, achieving a remarkable removal efficiency of 97% for RhB. SEM and TEM revealed a diverse morphology, an alginate fibrillar structure, Fe_2_O_3_ formed cubic or cuboid shapes, and Cu-MOF appeared as cubic crystals with sharp edges. Notably, distinct structures expose different crystal facets, which enhanced the adsorption process; this was further supported by studies investigating the effects of contact time, pH, and temperature.

Since material recyclability is a major challenge, Edirisooriya et al. conducted a detailed study on the recyclability and regeneration of Au/TiO_2_ and Pt/TiO_2_ [141]. They evaluated hydrogen production rates, turnover frequencies, and durability. Analysis of fresh, used, and regenerated catalysts showed that calcination-based regeneration greatly improved stability: Pt/TiO_2_ remained active for over 43 days, and Au/TiO_2_ for over 33 days across four cycles. The results suggest that catalyst deactivation was mainly caused by chemisorption, fouling, thermal degradation, sintering, and poisoning from synthesis residues and plastic byproducts. Moreover, it was observed that regeneration boosted catalytic activity by 20 times for Pt/TiO_2_ and 30 times for Au/TiO_2_. In the same context, Bockenstedt et al. proposed catalyst recovery, regeneration, and reuse, using gravity-assisted settling, centrifugation, and air plasma treatment [142]. The results showed that 77% of Aeroxide^®^ P25 TiO_2_ nanoparticles and 57% of porous TiO_2_ nanowire photocatalysts could be recovered and reused.

## 6. Design Strategies for Heterojunctions

A heterojunction is defined as the interfacial region between two semiconductors with compatible energy band structures. This interface can be formed either through direct bonding of the respective surfaces or through the alignment of internal crystal planes [143]. Historically, at least five types of heterojunctions have been reported, which are listed in Table 2. The former summarizes the main types of heterojunctions in semiconductor systems, including their band alignment, charge transfer mechanisms, advantages, and limitations, while emphasizing how the relative positions of the conduction and valence bands govern the migration and separation of photogenerated charge carriers. In general, type I heterojunctions favor the accumulation of both electrons and holes in the same semiconductor, which enhances recombination and is more suitable for light-emission applications rather than photocatalysis. Type II and p-n heterojunctions are widely used because they promote spatial charge separation through band offsets or internal electric fields. On the other hand, the Z-scheme and S-scheme heterojunctions have become the most widely studied in the 21st century. The Z-scheme photocatalytic system, which mimics natural photosynthesis, can be constructed using two semiconductors without the need for mediators, and its main distinction from other heterojunctions lies in the electron transfer pathway [144]. Such systems have been extensively employed for the photodegradation of hazardous molecules. For example, Co_0.08_Cd_0.92_S and Bi_2_MoO_6_ were combined to form a Z-heterojunction (Bi_2_MoO_6_/Co_0.08_Cd_0.92_S) and tested for the MB degradation, achieving an efficiency of 97.3%, which exceeded that of individual components [145]. The presence of both materials was confirmed by XRD, showing enhanced peak intensities and a peak shift attributed to the enlargement of the (100) planes. Successful heterojunction formation was further supported by XPS analysis, where shifts in binding energy of Bi 4f, O 1s, Mo 3d, Cd 3d, and S 2p indicated strong interaction between Co_0.08_Cd_0.92_S and Bi_2_MoO_6_, confirming the formation of the heterojunction. A recent study investigated a CeO_2_/CoFe_2_O_4_@g-C_3_N_4_ ternary heterojunction for the visible-light degradation of enrofloxacin (ENF) [146]. The materials were synthesized by a hydrothermal method followed by ultrasonication, and their formation was confirmed by P-XRD and FTIR. Photocatalytic tests revealed 98.25% ENF removal in 60 min, outperforming CeO_2_/CoFe_2_O_4_ (73.63%) and CoFe_2_O_4_ (57.30%).

The formation of an S-scheme heterojunction typically optimizes the band structure, facilitating charge-carrier separation and enhancing the spatial distribution of photogenerated electron-hole pairs [152]. A wide range of chemical elements, including noble and non-noble metals as well as rare earths, has been employed in the synthesis of these materials. For example, CdS was anchored onto CuO to form a CuO/CdS S-scheme heterojunction, which was applied for the selective conversion of CO_2_ to CO [153]. While CuO exhibits a blade-like morphology, CdS forms small particles that grow and subsequently attach to the surface of the CuO nanosheets. Formation of the S-scheme heterojunction alters the photoluminescence spectra of CuO/CdS, showing a reduced intensity compared to that of the individual components, indicating that the heterojunction effectively suppresses the recombination of photogenerated electron-hole pairs. Notably, the limited H_2_ evolution rate of merely 1.28 mmol g^−1^ h^−1^ in a water-glycerol mixture. In an S-scheme CdS/La_2_O_3_ system, enhanced by a factor of 14.5 times [154]. In addition, anatase/rutile TiO_2_ S-heterojunction has demonstrated solar-to-hydrogen efficiency (STH) of 14.25% at a pH of 0 and 7 [155]. The former efficiency is relevant considering that Liu et al. reported a STH of only 0.44% and an apparent quantum yield (AQY) of 9.13% using a β-NiS/TiO2-x Ohmic junction [156]. Recent reports on S-scheme photocatalysts further illustrate this disparity. For example, CdS/Co_3_S_4_ S-scheme nanoboxes can achieve hydrogen evolution rates as high as 23.45 mmol g^−1^ h^−1^ with an AQY of 18.5%, largely due to improved light harvesting and shortened charge transport pathways [157]. Similarly, other S-scheme systems such as NiO/CdS heterojunctions have demonstrated hydrogen evolution rates of 7.89 mmol g^−1^ h^−1^, about 24.7 times higher than pristine CdS, highlighting the strong role of interfacial band alignment and built-in electric fields in enhancing carrier separation [158].

A similar effect was reported in [159], where CdSe was used for the reduction in hexavalent chromium (Cr(VI)); the formation of a CeO_2_/CdSe heterojunction enhanced photocatalytic activity by approximately 1.77-fold. The authors attributed this enhancement to the three-dimensional micronetwork structure of CeO_2_, which provides a robust support for CdSe nanoparticles via strong interparticle interactions, facilitating intimate interfacial contact and promoting the formation of an efficient heterojunction. The enhancement of photocatalytic activity in S-scheme heterojunctions is governed by multiple factors, among which morphology plays a pivotal role. For instance, Lee et al. reported that the efficiency of antibiotic degradation over CdS/BiOIO_3_ S-scheme hybrid heterojunction is strongly influenced by the 1D/2D interfacial contact, which facilitates enhanced charge transfer [160].

CdS/N-rGO composites prepared via hydrothermal synthesis have also been proposed as S-scheme heterojunctions, demonstrating promising photocatalytic performance for H_2_ evolution with a notable efficiency of 58.8 mmol h^−1^g^−1^ under visible light irradiation [161]. A key observation was that no significant changes in the reflection peaks were observed in the nitrogen-doped composite (CdS/N-rGO) compared with the undoped CdS/rGO. However, incorporation of rGO or N-rGO significantly affected both the crystallite size and lattice parameters, which can be ascribed to the formation of chemical bonds. In the case of N-rGO, nitrogen atoms may interact with S or Cd species, thereby modifying bond lengths. SEM images revealed that pure CdS tends to agglomerate, whereas the addition of N-rGO resulted in reduced particle size and a more uniform distribution. These structural changes are consistent with the UV-vis measurements, which showed a redshift in the band gap to 2.27 eV. Although no structural defects were observed in this study, strain is known to play a vital role in heterojunction photocatalysts. For example, reference [162] reported lattice strain in Cu-MOF/ZFO composite as an indicator of heterojunction formation. Analysis of the Tauc plot revealed a reduced band gap of 1.98 eV for the Cu-MOF/ZFO composite, smaller than that of ZFO (2.26 eV) and Cu-MOF (2.17 eV), further confirming the successful heterojunction formation. A significant contribution was achieved by Xi and coworkers, who employed nanoarchitectonic frameworks to develop a nanohouse-like S-scheme heterojunction [163]. This material was constructed using NH_2_-MIL-125 as the MOF, which exhibits a plate-like morphology, onto which Co(OH)_2_ nanosheets and hollow ZIF-8 cages were subsequently grown. SEM images (Figure 8a–c) show a solid cake-like morphology of the final material. However, TEM images (Figure 8d–f) reveal that it is composed of distinct structural components, further confirmed by HAADF-STEM (Figure 8g) and elemental mapping analyses. The floor consists of NH2-MIL-125, on which Co(OH)_2_ preferentially grows, while ZIF-8 forms an enclosing shell. This spatial distribution is further supported by the line scan profile shown in Figure 8h. The reported band gap S-scheme heterojunction is 2.7 eV, significantly narrower than that of ZIF-8 (5.17 eV), thereby greatly enhancing the NH_4_^+^ production.

Beyond qualitative imaging techniques such as SEM or TEM, the characterization of heterojunction interfaces requires complementary spectroscopic and electrochemical methods to obtain quantitative information about interfacial interactions. Techniques such as XPS and ultraviolet photoelectron spectroscopy allow the determination of chemical states, band alignment, and work function differences between semiconductors, while photoluminescence (PL) and time-resolved PL spectroscopy provide insights into charge carrier recombination and lifetime. Additionally, electrochemical impedance spectroscopy (EIS) and transient photocurrent measurements can evaluate interfacial charge transfer resistance and carrier separation efficiency.

## 7. In Situ and Operando Techniques to Characterize the Photocatalyst

**Figure 9:**
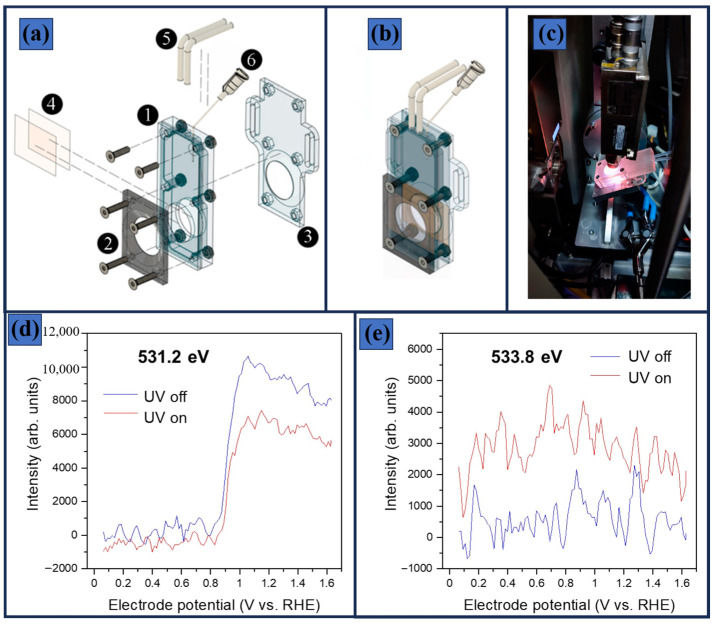
(**a**,**b**) Schematic diagram of the designed operando photocatalytic cell; (**c**) real image of the cell installed in the Rigaku R-XAS spectrometer. Reproduced under the terms and conditions of the Creative Commons Attribution (CC BY) license (https://creativecommons.org/licenses/by/4.0/) [164]; (**d**,**e**) XAS spectra for the TiO_2_ photocatalysts measured during a potential sweep at two energies at 531.2 eV and 533.8 eV. This article is reproduced under the terms of the Creative Commons CC-BY-NC license. © 2024 The Authors. Published by Elsevier B.V. [165].

The most effective approach to elucidating the mechanisms and hidden processes in photocatalysts is the use of advanced characterization techniques performed under in situ and operando conditions. Kozyr et al. [164] used X-ray absorption spectroscopy (XAS) to elucidate structural changes in Pt/TiO_2_ during the deposition process and under UV light irradiation for H_2_ generation. The spectra were recorded using an R-XAS Looper (Rigaku, Japan) in fluorescence yield geometry using a Si (620) Johansson bent monochromator (ΔE = 1.5 eV at the Pt L_3_-edge, 11,564 eV). The incident beam was monitored with an Ar-filled (300 mbar) ionization chamber, and fluorescence was detected by a silicon drift detector. Figure 9 presents the cell designed by this group, which consisted of two transparent Scotch tape windows separated by 4.6 mm. One window contained the photocatalytic material, while the other served as the entry point for light irradiation. A key experimental parameter was the XAS configuration, which operated in fluorescence mode. In this setup, the X-ray beam impinged on the sample at an incident angle of approximately 45°, while the detector was positioned at 90° relative to the beam to collect the emitted fluorescence signal. In a related study, conventional TiO_2_ was investigated under real-time and operando conditions using soft-XAS to monitor the oxygen evolution reaction (OER) photocatalyst surface [165]. The measurements were achieved in fluorescence-yield wavelength-dispersive XAS, where a Teflon electrochemical cell enabled probing of the solid–liquid interface, with the electrolyte separated from the vacuum by a 200 nm Si_3_N_4_ window (3 × 1 mm). Notably, during measurements conducted under a swept potential, pronounced peaks at 531.2 and 533.8 eV were observed only when the system was exposed to UV light. Figure 9d,e presents the corresponding XAS spectra acquired during the electrochemical test under a swept potential. In particular, the signal intensity in the 531.2 eV region increased markedly at potentials above 0.9 V vs. RHE, indicating potential-dependent modifications of the oxygen electronic states. These changes may be associated with the formation of photoinduced surface species or alteration in the TiO_2_ electronic structure under operando conditions.

The authors highlighted that XAS analysis was enabled by the use of wavelength-dispersive XAS, in which absorption across all energies is measured simultaneously and spectra are recorded continuously with acquisition times ranging from 0.1 to 10 s. In the same context, Abudukade et al. [166] studied the water-splitting process on Ni-SrTiO_3_ photocatalysts by using in situ XAS, with the in situ performed at PETRA III (beamline P65) at DESY, Hamburg, and at ELETTRA Sincrotrone Trieste, where a sealed liquid-phase cell with a 6 μm Mylar window was used to allow X-ray and UV transmission while enabling gas analysis. However, this group identified an important limitation of the XAS operating mode: during simultaneous exposure to X-rays and UV light, no changes in the Ni oxidation state were detected. In contrast, when the X-ray irradiation was applied intermittently, a clear transformation from Ni(0) to Ni(II) was observed, corresponding to the formation of NiO and Ni(OH)_2_ phases. In another study, in situ X-ray absorption fine structure (XAFS) spectroscopy was adopted to investigate the mechanism responsible for the photothermal activity of commercial WO_3_ powder under combined light and thermal conditions [167]. One advantage of photothermal catalysts is that thermal energy can promote the reaction, while light irradiation induces photocharge generation. Beyond the scientific insights provided by these studies, the authors also emphasized key challenges in the design of in situ cells, including the choice between single or dual-chamber configurations, the development of an efficient gas injection system, and the selection of thin films versus nanocatalysts. Catalyst thickness was identified as a critical parameter, as the incident light enters from the denser side of the material and may interact predominantly with inactive regions. To overcome this limitation, the use of ultrathin catalyst film or nanomaterials with a high surface-area-to-volume ratio was recommended to ensure improved light penetration and effective activation of catalytic sites. Additionally, the authors presented the final cell design along with photographs, dimensions, and construction details, which greatly facilitate the replication of in situ measurements. Another technique used is Bragg coherent X-ray diffraction imaging (BCDI) that reveals the displacement field of a material, which is directly related to lattice strain [168]. A representative example is the in situ characterization of Au nanoparticles supported on TiO_2_ films, which revealed wavelength-dependent photocatalytic strain evolution [169]. In this study, changes in the Au lattice were quantified, showing no variation under pristine conditions but a gradual lattice expansion under light irradiation: green light (0.0017–0.0019 Å), UV light (0.0027–0.0032 Å), and combined green/UV irradiation (0.0038–0.0043 Å). The authors established a correlation between lattice expansion and the generation of reactive oxygen species (ROS), with tensile strain increasing from ~0.2% under green light to ~0.3% under UV light and ~0.4% under combined green/UV irradiation. Furthermore, under green light illumination, electron transfer from Au to TiO_2_ was observed, leading to hole formations that promoted the generation of OH radicals. More recently, BCDI has been applied to investigate strain evolution in Bi_2_WO_6_ under operational conditions [170]. At 40 °C, charge carriers become activated, exhibiting increased mobility and suppressed recombination. During this transformation, a new phase nucleated near defect sites and propagated in a spatially heterogeneous matter. Moreover, small cracks formed as a result of local strain and environmental cycling, increasing the surface area and creating new reactive sites that may enhance material performance. These findings demonstrate that BCDI is a powerful technique for tracking structural and strain-related changes in photocatalysts under operando conditions.

In situ FTIR spectroscopy has demonstrated broad versatility across a wide range of material applications, from fuel cells to photoelectrochemical systems [171,172]. Palharim et al. employed this technique in attenuated total reflection (ATR) mode to monitor the degradation of acetaminophen on WO_3_-AgCl photocatalysts [173]. ATR-IR experiments were performed in a cooled copper flow-through cell (≈170 μL) with a quartz window and Viton O-ring. A ZnSe internal reflection element (IRE) coated with the photocatalyst was mounted in the cell and analyzed using a Bruker Vertex 80v equipped with an MCT detector. Spectra were recorded under vacuum at 4 cm^−1^ resolution with 200 scans. Illumination was provided by a 300 W Xe arc lamp. A key challenge was encountered during the measurement of a low acetaminophen concentration (100 mg L^−1^), where the FTIR signal was too weak for reliable analysis due to detection limits. As a result, the concentration was increased to 500 mg L^−1^ to obtain measurable spectra. However, this concentration is considerably higher than those typically used in photocatalytic degradation experiments, which may limit the environmental relevance of the results. In another work, the gas-phase degradation of 1-butanol and methanol was investigated on homemade TiO_2_ photocatalysts and a commercial Hombikat-b sample [174]. In the case of Hombikat-b, a decrease in the band at 1075 cm^−1^ was observed during the initial minutes of illumination, accompanied by the emergence of new bands at 1715, 1570, 1532, and 1440 cm^−1^. These bands were attributed to surface-adsorbed carboxylate intermediates, likely formed during the oxidative degradation of 1-butanol into species such as butanoates, acetates, and formates. The photocatalytic degradation of toluene has also been investigated using in situ FTIR spectroscopy on a CaFe_2_O_4_ hollow composite, where the technique proved effective in elucidating the reaction mechanism and identifying intermediate species as well as final products [175]. The results revealed a decrease in the characteristic toluene absorption bands under visible-light irradiation, while the intensities of the bands at 2361 and 2340 cm^−1^ increased, indicating CO_2_ formation and confirming the mineralization of toluene.

In situ environmental transmission electron microscopy (eTEM) has been implemented to directly visualize structural transformations in iron-based photocatalysts during CO_2_ reduction [176]. In this study, a δ-FeOOH photocatalyst was subject to stepwise heating under an H_2_ atmosphere, while structural evolution was monitored by in situ eTEM. At room temperature, the δ-FeOOH showed two dominant lattice planes ascribed to (100) and (110) reflections, with interplanar spacing of 0.253 and 0.147 nm, respectively. Upon heating above 200 °C, an additional lattice plane with a spacing of 0.271 nm emerged, corresponding to the (104) plane of α-Fe_2_O_3_, thereby indicating the onset of a thermally induced phase transformation. At 250 °C, the conversion of α-Fe_2_O_3_ to Fe_3_O_4_ commenced and progressed with increasing temperature, reaching completion at approximately 300 °C. Further heating to 400 °C resulted in the exclusive presence of α-Fe, confirming complete reduction to metallic iron under the reducing H_2_ environment.

## 8. Computational Modeling and DFT Simulations, and Machine Learning Design

Based on practical experience, experimental development of photocatalysts can be both time-consuming and costly, slowing progress in the design of efficient nanostructures. In this context, computational modeling has increasingly been adopted to elucidate the mechanisms involved during both the synthesis process and the degradation of target pollutants. Atomic/scale modeling based on density functional theory (DFT) is a widely employed computational approach for understanding and predicting materials performance at the atomic level [177]. This methodology accounts for key parameters such as thermodynamic energies, bond lengths, atomic radii, electronic band structures, and density of states (DOS), among others [178]. The aforementioned factors are employed to interpret the structural, electronic, and catalytic properties of materials. Among the most extensively used computational platforms is the Vienna ab initio Simulation Package (VASP), which permits the specification of key parameters such as the exchange-correlation functional, plane-wave cutoff energy, k-point sampling within the Brillouin zone, and pseudopotentials for electron-ion interactions [179]. In addition, VASP supports the inclusion of spin polarization, Hubbard U corrections, and van der Waals interactions, allowing for accurate modeling of electronic structure, total energies, band gaps, and other critical material characteristics of a broad range of systems.

**Figure 10:**
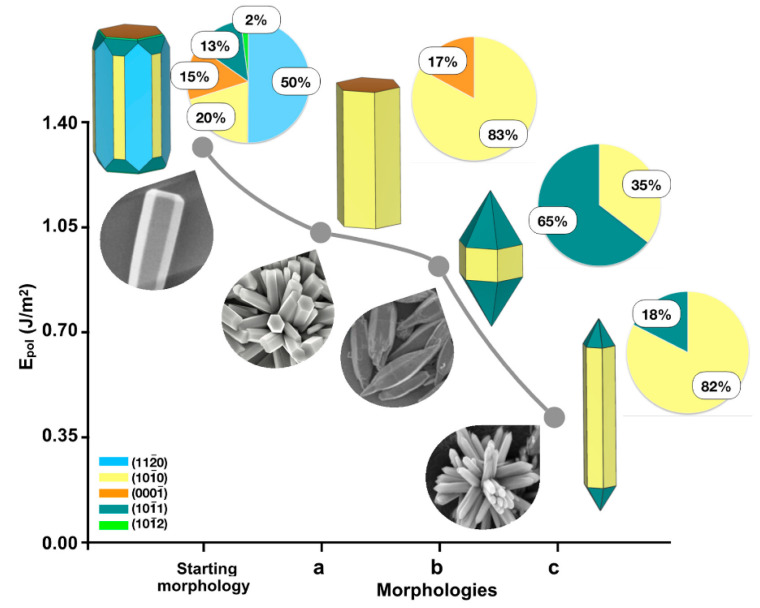
Analysis of polyhedron energy E_pol_ depending on morphologies. Reproduced under the terms and conditions of the Creative Commons Attribution (CC BY) license (https://creativecommons.org/licenses/by/4.0/) [181].

In a systematic study, Ribeiro et al. theoretically correlated the surface structure and morphology of Ag_2_O photocatalysts [180]. First-principles calculations demonstrated that the local coordination environment and electronic configuration of Ag species are responsible for the observed enhancement in photocatalytic activity, with electrons preferentially migrating to the (111) facets and holes to the (100) and (110) facets. Notably, these crystallographic planes in Ag_2_O are strongly influenced by particle shape and size, with cubic, octahedral, rhombic, dodecahedral, and rhombicuboctahedral morphologies identified as particularly favorable for achieving superior photocatalytic performance. The authors constructed a plot correlating the polyhedron energy (E_pol_) with three distinct reaction pathways for Ag_2_O. For reaction pathways A and B, the most stable morphologies were rhombicuboctahedral and cubic structures, which expose a higher proportion of (100) and (110) facets, thereby enhancing photoactivity. In contrast, when the relative contribution of the (111) surface increased with respect to (100) facets, a decrease in photocatalytic activity was observed. The dependency of materials properties on surface structure was further systematically investigated by Gouveia et al. [181], who developed an Epol-reaction pathway plot based on various morphologies reported in the literature. As can be seen from Figure 10, distinct tetradecahedral ZnO morphologies can be obtained by tuning the synthesis parameters, which in turn modulate the reaction pathway. The sample denoted as the initial stage was produced via a low-temperature hydrothermal process in the absence of surfactants, resulting in an early-growth ZnO structure. In contrast, the material exhibiting morphology-a (an elongated hexagonal form) was synthesized using the same hydrothermal synthesis route but with adjusted pH, increased temperature, and prolonged reaction time—conditions that promoted further crystal growth and facet development. Furthermore, morphologies -c and -d were obtained using a mixed water/alcohol solvent system, demonstrating the critical role of solvent composition in controlling nucleation dynamics and final particle morphology.

Huang et al. [182] employed DFT to investigate the effects of vacancies on the properties of Bi_4_O_5_Br_2_ composites. Their results demonstrated that the formation of oxygen or bromine vacancies is energetically more favorable than the formation of bismuth vacancies, the latter inducing only minor lattice distortion. Moreover, the presence of oxygen vacancies was found to narrow the band gap, which enhances visible/light harvesting and promotes more efficient charge carrier transport. These findings are in good agreement with the DFT results reported by Katai et al., who observed that oxygen vacancies in black TiO_2_ brookite reduce the optical band gap and enhance CO_2_ adsorption [183]. Similarly, DFT studies were conducted to examine oxygen vacancies and charge trapping on (101) and (001) surfaces, as well as in Ti_33_O_33(2−δ)_ and Ti_151_O_151(2−δ)_ nanoparticles [184]. In the case of nanoparticles, electrons associated with oxygen vacancies tend to localize Ti^3+^ species, which can be further reduced to Ti^2+^ under strongly reducing conditions. Ti^3+^ species were detected by electron paramagnetic resonance (EPR), while Ti^2+^ acts as a hidden charge trap. The authors stated that particle size significantly influenced reducibility, with smaller nanoparticles being more favorable for photocatalytic applications due to enhanced visible-light absorption and increased surface area. Saroar et al. [185] combine DFT calculations with experimental measurements to determine the electronic, optical, and phonon properties of γ-Bi_2_MoO_6_. The theoretical calculations accurately reproduced experimentally measured Raman and infrared spectra, confirming the power of DFT simulations. Meanwhile, the authors implement the Heyd-Scuseria-Ernzerhof (HSE06) hybrid functional, including van der Waals interactions and relativistic spin–orbit coupling corrections, to predict the band gap, which closely matched values obtained from diffuse reflectance spectroscopy. More complex materials have also been investigated using DFT, such as the double perovskite RbBa_2_Ti_3_O_10_, to gain insight into its structural, mechanical, and electronic properties [186]. This theoretical investigation revealed that the material exhibits a band gap of 1.66 eV and shows potential for water splitting processes.

Molecular dynamics (MD) is another computational approach used to simulate the photoactivity of target materials, helping to address uncertainties associated with surface reaction mechanisms. A comprehensive investigation of ZnO nanostructures, combining experimental and theoretical methods to elucidate morphology-dependent photoactivity and reaction pathways [187]. Experimentally, distinct morphologies were obtained by varying the Zn:KOH ratio, with nanostar-like structures exhibiting the highest photocatalytic activity toward methylene blue (MB) degradation. Complementarily, MD simulations were employed to investigate the interactions between oxygen species and MB, providing atomistic insight into the underlying reaction mechanism. In a similar study, Haounati et al. [188] combined MD simulations and experimental analysis to evaluate ZnO supported on Montmorillonite clay as a photocatalyst for rhodamine B (RhB) degradation. The theoretical simulations indicated that RhB adsorption onto the catalyst surface is thermodynamically favorable, as evidenced by the negative interaction energies associated with the exothermic adsorption process.

Beyond first-principles and molecular dynamics simulations, machine learning (ML) has gained significant attention as a consequence of digital transformation and advances in algorithm development, emerging as a powerful tool for addressing complex engineering problems [189]. Herein, it is necessary to highlight that ML models and DFT calculations differ in approach and purpose. ML models are data-driven tools that learn patterns from existing datasets to rapidly predict material properties and catalytic performance, while DFT calculations are first-principles quantum mechanical methods that describe the electronic structure of materials and provide mechanistic insights into processes. In this context, Li et al. [190] adopted dynamic ML to optimize microwave-assisted synthesis parameters, using quercetin loading, microwave irradiation time and power, H_2_SO_4_ volume, and ethanol volume as input variables, with catalyst yield as the output. Real-Time Guidance ML framework demonstrated robust predictive performance despite the limited size of the dataset. Guided by a trained model, the optimized photocatalyst was synthesized experimentally, achieving an H_2_O_2_ production rate of 11,544 μmol g^−1^ h^−1^. In a separate systematic study, ML-driven design strategy for covalent triazine frameworks was reported and subsequently validated through experimental synthesis and characterization, highlighting the potential of an ML-assisted material discovery in photocatalysts [191]. An orthogonal group SO(3)-invariant graph neural network was utilized as a predictor for the photocatalytic activity of the materials. The DimeNet++ (Directional Message Passing Neural Network++)-based model analyzed 14,920 structures and identified 45 potential candidates, achieving high predictive accuracy with an R^2^ value of 0.98. A notable strength of this model is its ability to simultaneously account for thermodynamic stability, the Perdew-Burke-Ernzerhof (PBE) band gap, band edge positions, and synthesizability. Within these ML frameworks, an Mn_3_O_4_-based photocatalyst has also been fabricated [192]. In addition to the band gap, determined from density functional theory, the effective mass was also used as a descriptor to obtain the optimal Al content in the Al_x_Mn_3-x_O_4_/Ag_3_PO_4_ heterojunction. Interestingly, the model was trained using the *C/C*_0_ degradation rate measured after 2 h, where *C* represents the concentration of the MB solution, and *C*_0_ is its initial concentration. The dataset consisted of 30 materials synthesized via the sonication-assisted co-precipitation method with varying *x* and *m* values. ML optimization enables the identification of Al_0.5_Mn_2.5_O_4_/35 wt.% Ag_3_PO_4_ as the optimal composition, exhibiting a 27-fold enhancement.

**Figure 11:**
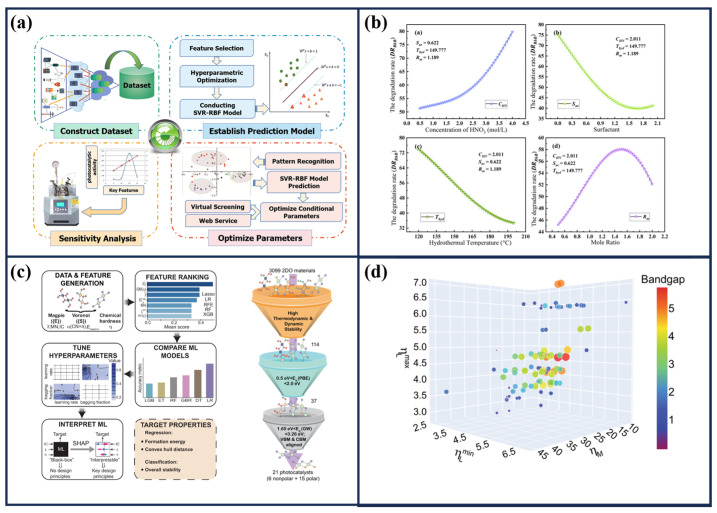
(**a**) Complete workflow of the ML analysis for designing MLM-BWO/MIL. (**b**) Sensitive analysis of each key feature determined by ML. Reproduced under the terms of the Creative Commons Attribution-NonCommercial 3.0 Unported License [193]. (**c**) Diagram for the applied Shapley Additive exPlanations (SHAP) ML model to determine efficient 2D water-splitting photocatalysts. (**d**) Band gap variation of 2D materials according to the Perdew-Burke-Ernzerhof (PBE) function. Reproduced under a Creative Commons Attribution 4.0 International License (http://creativecommons.org/licenses/by/4.0/) [194].

A comprehensive study by Zhai et al. examined 13 variables across a dataset of 53 samples, which were analyzed by support vector regression (SVR) to design Bi_2_WO_6_/MIL-53(Al) nanocomposites [193]. In this work, a forward feature selection method was integrated into the SVR algorithm to identify key variables, while a radial basis function combined with virtual screening was used to determine the optimal synthesis parameters. The complete steps of the ML process are depicted in Figure 11a, beginning with dataset construction and concluding with sensitivity analysis. The ML analysis revealed that only four parameters were most critical for enhancing RhB degradation: the molar ratio of Bi_2_WO_6_ to MIL-53(Al), hydrothermal temperature, concentration of HNO_3_, and surfactant type. Figure 11b presents the individual effects of the four main parameters (concentration of HNO_3_, surfactant, hydrothermal temperature, and mole ratio) affecting the degradation rate. Among them, HNO_3_ concentration exhibits a positive correlation with degradation efficiency, indicating that higher concentrations of HNO_3_ lead to enhanced degradation performance. Although the implemented ML results in good precision, it can be observed that more complex ML with multiple layers has been adopted to further analyze large datasets. Figure 11c shows the ML workflow reported in [194], where more than 100 features were initially generated. To improve computational efficiency, a streamlined feature selection process was implemented using Random Forests (RFs), linear regression (or logistic regression for classification), least absolute shrinkage and selection operator (LASSO), recursive feature elimination (RFE), and extreme gradient boosting (XGBoost). Finally, Figure 11d presents the influence of ligands bonded to the metal (ηL) and the Pearson chemical hardness of the metal cation (ηM) on the obtained bandgap.

An important finding reported in [193] was the correlation between morphology and the combined effects of hydrothermal temperature and surfactant type, which led to the formation of 2D nanosheets. These results highlight the capability of ML to guide materials engineering by enabling control over morphology and structural properties to enhance photocatalytic activity. In this sense, a Gaussian process regression (GPR) model was adopted to predict the band gap of anatase TiO_2_, considering surface area and lattice parameters as descriptors [195]. A total of 60 samples were used to develop the GPR model in MATLAB, and its performance was evaluated using the root-mean-square error (RMSE) and mean absolute error (MAE). The exponential kernel with a constant basis function demonstrates good performance, yielding RMSE and MAE values of 0.0012 and 0.0010%, respectively. Initially, the authors focused on the effect of metal and non-metal ion incorporation into the TiO_2_ structure, which led to changes to lattice parameters and surface area. However, as highlighted in previous ML studies, even seemingly basic factors such as surfactants can influence crystal nucleation and growth.

**Figure 12:**
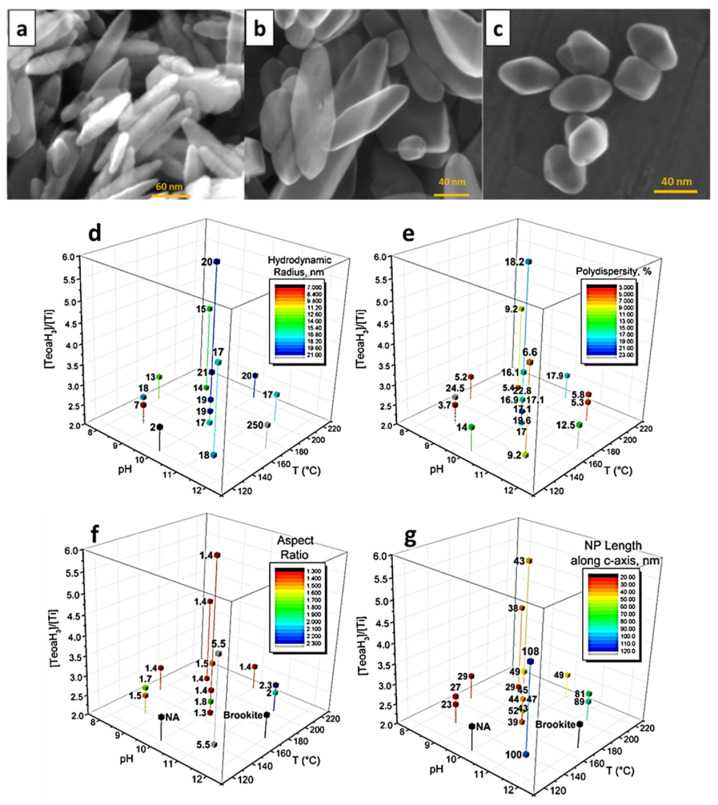
(**a**–**c**) SEM images of samples HT06, HT08, and HT16, selected as three representative cases from a total of 20 samples analyzed according to the experimental design. (**d**–**g**) Effect of the 4 factors on each output parameter. Reproduced under a Creative Commons Attribution 4.0 International License (http://creativecommons.org/licenses/by/4.0/) [196].

An insightful study by Pellegrino et al. [196] employed ML to investigate the effect of triethanolamine, titanate (TeoaH_3_), initial pH, and operating temperature on the hydrothermal synthesis of TiO_2_ nanoparticles. The experimental design was based on a Box-Wilson central composite design that considers the four aforementioned parameters, with outputs focused on hydrodynamic radius, polydispersity, and aspect ratio. Figure 12 summarizes the effect of each factor on the final properties of the TiO_2_. From these results, it can be inferred that particle elongation is favored by high pH values across the entire temperature range. A similar trend is observed with increasing TeoaH_3_ concentration, further demonstrating that morphological changes are influenced by these synthesis parameters.

A more effective strategy combining first-principles calculations with ML was published by Chen et al., who investigated g-C_3_N_4_ combined with 3d transition metals as a potential photocatalyst for nitrogen fixation [197]. Herein, first-principles calculations were employed to determine intrinsic material properties such as light absorption, while ML techniques, including the backward elimination method, sure independence screening, and sparsifying operators, were used as advanced tools to narrow down and predict candidate materials with superior performance beyond conventional theoretical screening.

The Vienna ab initio simulation package was configured to model a g-C_3_N_4_ supercell containing 48 carbon atoms and 64 nitrogen atoms, with a Brillouin zone sampling performed using a 1 × 1 × 1 k-point grid centered at the γ(G) point. Although this integrated approach enables the identification of optimal materials even with limited datasets, a major obstacle remains the high level of expertise required from both researchers and practitioners. Table 3 presents a summary of representative works that applied machine learning to evaluate the degradation capability of photocatalysts.

## 9. Challenges and Future Perspectives

Despite considerable progress in photocatalyst development, several challenges continue to hinder their large-scale application. One major issue is the scalability of current synthesis strategies. Many widely used laboratory methods, such as hydrothermal, solvothermal, and template-assisted routes, often require strict control of temperature, pressure, and precursor concentrations, which complicates their translation to industrial-scale production. In large-scale systems, mass and heat transfer limitations, concentration gradients, and non-uniform nucleation conditions can significantly affect crystal growth, resulting in reduced reproducibility and inconsistent material properties. Therefore, scalable synthesis strategies such as continuous-flow hydrothermal reactors, flame spray pyrolysis, spray drying, and microreactor-based synthesis have been increasingly explored to enable better control over nucleation and growth. Additionally, green and cost-effective approaches, such as solvent-free methods, low-temperature synthesis, and the use of abundant precursors, are essential to reduce energy consumption and environmental impact during industrial production. Consequently, systematic investigations are needed to determine whether the structural, morphological, and electronic properties of photocatalysts can be preserved during scale-up while maintaining cost-effectiveness and reproducibility.

Another critical challenge concerns the long-term stability and recyclability of photocatalysts. Under prolonged irradiation and reactive environments, photocatalytic materials may undergo photocorrosion, structural degradation, or surface fouling, leading to a gradual loss of activity. Although some authors report the recyclability of photocatalysts via thermal treatment and air plasma, most studies lack a comprehensive cost-effectiveness analysis. Moreover, most reported photocatalytic studies are performed under controlled laboratory conditions using model pollutants and idealized light sources. However, real environmental systems often contain complex mixtures of contaminants, variable pH, and competing ions that can significantly affect catalytic performance. Future research should therefore focus on evaluating photocatalysts under realistic conditions, including natural sunlight irradiation and continuous-flow systems.

Another critical concern is the potential risk of secondary contamination, as some nanoparticle-based photocatalysts may partially dissolve in water, thereby introducing new pollutants. Furthermore, the complexity of real wastewater, which often contains multiple coexisting contaminants, significantly reduces photocatalytic efficiency. This reduction arises from competitive adsorption at active sites, light attenuation, and the scavenging of reactive oxygen species.

To address these challenges, future research should focus on designing photocatalysts based on earth-abundant and non-toxic chemical elements, as well as developing robust immobilization and recovery strategies. Importantly, these approaches must be evaluated under realistic wastewater conditions to generate reliable data for the material. In this regard, ML and modeling offer useful tools to accelerate catalyst development by enabling rapid predictions of catalyst behavior, identification of key governing parameters, and reduction in experimental workload.

## 10. Conclusions

This work presents a clear correlation between morphology, structure, and photocatalytic activity, demonstrating that precise control over parameters such as capping agents, solvent selection, synthesis conditions, and heterojunction engineering can optimize photocatalytic performance. According to the revision, hydrothermal and solution-based synthesis methods remain the most widely adopted due to their versatility, relatively low cost, and ability to produce a wide range of nanostructures. Furthermore, it is necessary to highlight that parameters such as temperature, pH, solvent composition, precursor concentration, and the presence of surfactants or capping agents strongly influence nucleation and crystal growth processes, which are governed by several mechanisms, including Ostwald ripening, oriented attachment, and surface energy modulation.

On the other hand, the adoption of tailored architectures, such as quantum dots, one-dimensional nanowires, and hierarchical structures, along with defect engineering, facet exposure, and the incorporation of heterostructures or supporting matrices, can be a reliable strategy to enhance light absorption, increase surface area, and promote efficient separation and transport of photogenerated charge carriers. Finally, the integration of in situ and operando characterization techniques with computational approaches, particularly density functional theory, is positioned as a powerful strategy for understanding the structure–property relationships governing photocatalytic activity, as these combined experimental and theoretical efforts provide valuable guidance for the rational design of advanced photocatalysts. Further studies are essential to evaluate the long-term stability, reusability, and industrial viability of these photocatalysts, as most current reports overlook cost-effectiveness and the potential environmental impact of regeneration methods such as thermal recycling or plasma treatment.

## Figures and Tables

**Table 1:** Overview of widely used photocatalyst synthesis methods and critical parameters.

Material	Synthesis Process	Conditions	Solvent and Caping Agent	Morphology	Ref.
Ce_1-(3/4)x_Eu_x_O_2_	Microwave-assisted hydrothermal	8 min irradiation to reach 100, 140, 180, or 200 °C.	DI water, 2, 0.06, or 6 mol L^−1^, NaOH solution.	Nanorods and nanocubes.	[54]
TiO_2_	Sol-gel	Stirring for 24 h at 25 °C and 60 °C. Calcined at 450 °C for 4 h, 1 °C min^−1^.	DI water and ethanol. SDS, CTAB, and PEG.	Spherical, semi-spherical and worm-like shapes.	[63]
Bi_2_WO_6_	Hydrothermal	Hydrothermal for 15 h at 180 °C.	DI water and 20 mL of HNO_3_, NaOH solution.	Nanoplates.	[55]
WO_3_	Solvothermal	Heated at 120 °C for 12 h.	Acetone: DI water, 2:5 *v*/*v*, oxalate or citric.	Nanospheres and nanocages.	[57]
KBi_6_O_9_I/Ag-AgVO_3_	Combustion synthesis/sonochemical	At 80 °C, the gel was obtained, heated at 300 °C, sonicated for 45 min, and treated at 180 °C for 24 h.	DI water	Irregular polygon-like surface structure.	[64]
Co(II)-Pyridine-Decorated	Hydrothermal	Reagent dissolved in H_2_O and pH adjusted by H_3_PO_4_ and NaOH, heated in a reactor at 160 °C for 4 days.	DI water	Unevenly shaped blocks.	[65]
Bi_2_O_3_-Bi_2_SiO_5_	Sol-gel	Reagents were mixed with the solvents, and then the gel was calcinated at 400 °C for 2 h or 450 °C for 4 h.	Ethylene glycol, tetraethoxysilane.	Nanoparticles	[66]
BiOBr	Solvothermal and one-pot method	The Bi-reagent was dissolved in ethylene glycol, and NaBr was added under stirring and heated at 170 °C for 6 h.	Ethylene glycol	Flower-like structure.	[67]
Fe@Co-CP-2	Hydrothermal method and ion-exchange	Reagents were mixed with NaOH solution and heated at 130 °C for 4 days. Ion-exchange was achieved by mixing the obtained samples in a methanol solution.	H_2_O, methanol solution.	Irregular polyhedral.	[68]
NiS_2_/CdS	Hydrothermal synthesis + Ultrasonic-assisted composite synthesis	H_2_O_2_-CdS was dissolved in H_2_O_2,_ DI water, and put in an autoclave at 180 °C for 10 h. For NiS_2_ the chemicals were dissolved in DI water and reacted at 80 °C for 12 h.	DI water, H_2_O_2_, and ethanol.	Cone shape to mesoporous particles.	[69]
TiO_2_/MoSx/Ag	Photodeposition	TiO_2_/a-MoSx: TiO_2_ nanoparticles in DI water, then (NH_4_)_2_MoS_4_ in ethanol, was added and exposed to a Xe lamp. Ag nanoparticles: AgNO_3_ in ethanol was added and exposed to the Xe lamp.	DI water, ethanol	Nanoparticles.	[70]
La-doping ZnO	Co-precipitation	Reagents dissolved in DI water, NaOH solution was added until the pH was 12. Then calcinated for 2 h at 500 °C.	DI H_2_O	Disrupted flower-like morphology.	[71]

**Table 2:** Summary of heterojunctions along with their main characteristics.

Heterojunction	Band Alignment	Charge Transfer Mechanism	Advantages	Disadvantages	Ref.
Type I heterojunction (straddling gap).	CB and VB of one material straddle between the bands of the other.	Both electrons and holes migrate into the narrower bandgap semiconductor.	Strong recombination process, useful for light emission.	Poor charge separation, not ideal for photocatalysis.	[147]
Type II heterojunction (staggered gap).	CB of one is lower, VB of the other is higher.	Electrons and holes are spatially separate in different materials.	Efficient charge separation process.	Reduced redox potential due to charge loss.	[143]
p-n heterojunction.	Fermi levels align at the interface, forming a depletion region.	Built-in electric field drives the separation of electrons and holes.	Simple design, strong internal field, scalable.	Recombination at the interface is possible.	[148,149]
Z-scheme heterojunction.	Resembles natural photosynthesis.	Only high-energy electrons and holes are retained.	Strong redox ability mimics a natural process.	Requires mediator (redox pair, solid bridge).	[150]
S-scheme heterojunction.	Band bending leads to selective recombination at the interface.	Unfavorable electrons and holes recombine, leaving the most energetic ones.	High redox ability, strong charge separation, no mediator needed.	More complex design, less explored than other materials.	[151]

**Table 3:** Representative studies using ML to analyze morphological and structural features of photocatalysts and predict photodegradation performances.

Material	Algorithm/Dataset	Remarks	Considerations	Ref.
Covalent triazine frameworks	DimeNet++/14,920 CTFs structures.	R2 > 0.98 and MAE < 0.008 eV or 0.0014 eV/atom.	Thermodynamic stability, Perdew-Burke-Ernzerhof band gap, and band edge positions.	[191]
Al_0.5_Mn_2.5_O_4_/35 wt. % Ag_3_PO_4_	RF, EXT, GBR, KRR, and SVR/30 materials.	For EXT R^2^ = 1.00	Effective mass, band gap, and degradation efficiency.	[192]
Bi_2_WO_6_/MIL-53(Al)	SVR/53 samples	R = 0.823 for the degradation rate of RhB dye.	Mole ratio, temperature, concentration of HNO_3_, surfactant, and nine other variables.	[193]
TiO_2_	GPR model/60 samples	RME: 0.0012%; MAE 0.0010%.	Surface area and lattice parameter.	[195]
TiO_2_	Artificial Neural Network and Genetic algorithm/20 samples.	Not defined	Triethanolamine, titanatrane (TeoaH_3_), initial pH, and operating temperature.	[196]
ZnTe-based alloys	Sure, independence screening, sparsifying operator, and the agreement approach/13 ternary component.	RMES	The lattice constant, the equilibrium temperature of the compounds, and the band edge positions.	[198]

## Data Availability

No new data were created or analyzed in this study. Data sharing is not applicable to this article.
